# Phytochemistry, Pharmacological Potential, and Ethnomedicinal Relevance of *Achillea nobilis* and Its Subspecies: A Comprehensive Review

**DOI:** 10.3390/molecules30112460

**Published:** 2025-06-04

**Authors:** Anastassiya Shevchenko, Aiman Аkhelova, Shamshabanu Nokerbek, Aigul Kaldybayeva, Lyazzat Sagyndykova, Karlygash Raganina, Raushan Dossymbekova, Aliya Meldebekova, Akerke Amirkhanova, Yerbol Ikhsanov, Gulzhan Sauranbayeva, Manshuk Kamalova, Aidana Toregeldieva

**Affiliations:** 1 Higher School of Medicine, Al-Farabi Kazakh National University, Tole-bi 96, Almaty 050040, Kazakhstan; shevchenko.anastassiya@med-kaznu.com (A.S.); kamalova.manshuk@med-kaznu.com (M.K.); toregeldiyeva.aidana@med-kaznu.com (A.T.); 2 Department of Chemistry and Technology of Organic Substances, Natural Compounds and Polymers, Faculty of Chemistry and Chemical Technology, Al-Farabi Kazakh National University, Almaty 050040, Kazakhstan; erbol.ih@gmail.com; 3 Department of Pharmaceutical and Toxicological Chemistry, S.D. Asfendiyarov Kazakh National Medical University, Tole-bi 94, Almaty 050012, Kazakhstan; aigul_240873@mail.ru (A.K.); sauranbaeva.g@kaznmu.kz (G.S.); 4 School of Pharmacy, S.D. Asfendiyarov Kazakh National Medical University, Tole-bi 94, Almaty 050012, Kazakhstan; shamshabanu.nokerbek@mail.ru; 5 Department of Pharmaceutical Disciplines, Astana Medical University, Saryarqa Ave., 33, Astana 010000, Kazakhstan; sagyndykova.l@amu.kz; 6 Department of Pharmaceutical Technology, S.D. Asfendiyarov Kazakh National Medical University, Tole-bi 94, Almaty 050012, Kazakhstan; raganina.k@kaznmu.kz; 7 Department of Biochemistry, S.D. Asfendiyarov Kazakh National Medical University, Tole-bi 94, Almaty 050012, Kazakhstan; dos.raushan@mail.ru; 8 Department of Biotechnology, Faculty of Biology and biotechnology, Al-Farabi Kazakh National University, Almaty 050040, Kazakhstan; 9 Department of Biotechnology and General Chemical Engineering, School of Pharmacy, S.D. Asfendiyarov Kazakh National Medical University, Tole-bi 94, Almaty 050012, Kazakhstan

**Keywords:** ethnomedicine, phytochemistry, flavonoids, monoterpenes, pharmacological activities, antibacterial, antinociceptive

## Abstract

*Achillea nobilis* and its subspecies (*A. nobilis* subsp. *neilreichii* and *A. nobilis* subsp. *sipylea*) have been traditionally used in various ethnomedical systems across Eurasia. However, comprehensive studies on their phytochemical composition and pharmacological properties are still insufficient. This review aims to provide a critical synthesis of current knowledge regarding the botanical characteristics, geographic distribution, traditional applications, chemical constituents, and pharmacological effects of *A. nobilis* A structured search was conducted using eight scientific platforms, including Scopus, PubMed, Web of Science, Google Scholar, Science.gov, ScienceDirect, JSTOR, and BASE. Keywords related to phytochemistry, pharmacology, and ethnomedicine were applied, and a total of 28,000 records were initially retrieved. After a multi-stage screening process based on inclusion and exclusion criteria, 167 peer-reviewed publications from 1952 to 2023 were selected for detailed evaluation. Findings reveal a diverse range of bioactive compounds, such as flavonoids, monoterpenes, sesquiterpenes, and sesquiterpene lactones, which demonstrate antioxidant, antimicrobial, anti-inflammatory, antinociceptive, antispasmodic, and anticonvulsant activities. Most studies have focused on aerial parts and water-based extracts, while the root chemistry and organ-specific metabolite profiles remain largely unexplored. This review highlights the therapeutic potential of *A. nobilis* and underscores the need for future studies using multi-omics and advanced analytical techniques to support its development in pharmaceutical and nutraceutical applications.

## 1. Introduction

The exploration of specific plant species with ethnomedicinal value has proven instrumental in identifying novel bioactive compounds for therapeutic use [1]. *Achillea nobilis* L. and its subspecies (*A. nobilis* subsp. *sipylea*, *A. nobilis* subsp. *neilreichii*, and *A. nobilis* subsp. *nobilis*) represent a group of aromatic and medicinal plants widely used in traditional medicine systems across Eurasia [2,3,4]. These taxa belong to the Asteraceae family and have been historically used in herbal decoctions, infusions, oils, and topical applications aimed at treating pain, inflammation, gastrointestinal discomfort, respiratory infections, and dermatological conditions [5,6,7,8,9,10]. Their longstanding therapeutic applications are rooted in folk medicine systems from Turkey, Iran, Bosnia and Herzegovina, and Kazakhstan, among others [11,12,13,14,15].

**Figure 1:**
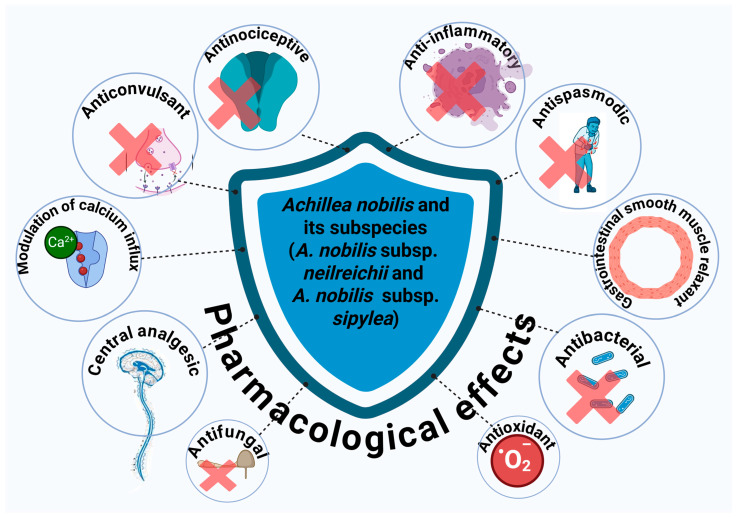
Pharmacological effects of *A. nobilis* and its subspecies.

Despite their documented use in traditional medicine, comprehensive scientific analysis of their phytochemical composition and pharmacological properties remains fragmented [16,17]. Reports suggest that *A. nobilis* and its subspecies contain a diverse array of bioactive secondary metabolites, including flavonoids, flavonoid glycosides, monoterpenes, sesquiterpenes, sesquiterpene lactones, and terpenoid derivatives [18,19,20,21,22,23,24,25,26,27,28,29,30,31,32,33,34]. These compounds have been linked to various biological activities, particularly antimicrobial, anti-inflammatory, antispasmodic, and neuroprotective effects. For example, flavonoids such as apigenin and luteolin glycosides exhibit strong antioxidant and anti-inflammatory properties, while volatile monoterpenes such as α-thujone, cineole, and camphor have shown antimicrobial activity (Figure 1) [21].

Phytochemical investigations from 1978 to 2020 have primarily focused on aerial parts of the plant and water-soluble extracts, often overlooking the phytochemical diversity of underground organs and lipid-soluble fractions. This creates a significant knowledge gap in understanding the complete metabolic profile of the species. Furthermore, studies suggest that the chemical profiles of different plant parts and subspecies vary significantly due to genetic and ecological factors, necessitating chemotaxonomic approaches and organ-specific metabolite profiling [27,28]. Seasonal variations have been reported to influence the concentration of essential oils and flavonoid content, with certain bioactive compounds peaking during flowering or specific phenological stages. Similarly, regional differences, including altitude, soil composition, and climatic conditions, can significantly modulate metabolite accumulation, leading to variability in pharmacological potency across populations [2,20,25]**.** However, these differences have not yet been fully characterized in a systematic manner.

Although several review articles have addressed the phytochemistry and pharmacological properties of the *Achillea* genus more broadly [4,35,36,37], none have provided a comprehensive, species-specific focus on *Achillea nobilis* and its subspecies. Existing reviews such as Barda et al. [4] and Saeidnia et al. [37] compile data primarily for *A. millefolium* and other well-studied species, while limited attention is given to the distinct phytochemical profiles and ethnomedicinal uses of *A. nobilis* taxa. Furthermore, no review has yet systematically explored the chemotaxonomic distinctions among *A. nobilis* subspecies or the variation in lipophilic and underground organ-derived metabolites. This review aims to fill this gap by offering a dedicated analysis of *A. nobilis* and its subspecies, integrating phytochemical, ethnopharmacological, and pharmacological data, and proposing future research directions using multi-omics and regional comparative approaches.

Recent advances in plant metabolomics, transcriptomics, and chemoinformatics offer the opportunity to resolve these limitations [29,30,31,32,33]. Future research should incorporate integrative multi-omics strategies to explore biosynthetic pathways, quantify metabolite variation across plant parts and environments, and establish pharmacological correlations with specific compounds. Additionally, understanding the influence of geographical and seasonal variability on phytochemical expression will enhance the reproducibility and quality control of derived herbal products. Given these gaps, a focused and comprehensive review is essential to consolidate current knowledge and guide future investigations.

This review aims to offer a thorough and cohesive summary of *A. nobilis* and its subspecies by consolidating existing information on their botanical traits, geographic distribution, traditional medicinal applications, phytochemical compositions, and pharmacological effects. The objective is to bridge traditional ethnobotanical insights with contemporary phytochemical and pharmacological evidence while identifying research gaps related to underexplored plant parts such as roots. Furthermore, the review proposes future research directions using advanced analytical and systems biology tools to support the safe and effective integration of *A. nobilis* into pharmaceutical and nutraceutical development.

## 2. Methods

A comprehensive and systematic literature review was conducted to identify relevant scientific publications pertaining to *Achillea nobilis* and its subspecies. The search aimed to capture a wide spectrum of studies addressing botanical, phytochemical, ethnomedicinal, and pharmacological aspects. Eight databases were strategically selected to ensure broad and high-quality coverage of the literature. These included bibliographic databases (Scopus, PubMed, Web of Science), academic search engines (Google Scholar, Science.gov), and full-text repositories (ScienceDirect, JSTOR, BASE), which collectively provide access to peer-reviewed journals, open-access materials, institutional repositories, and preprints.

To refine the search and extract relevant literature, targeted keywords were employed, including “*A. nobilis*”, “*A. nobilis* phytochemicals”, “*A. nobilis* compounds”, “*A. nobilis* pharmacological”, “*A. nobilis* traditional use”, and “*A. nobilis* ethnomedicine”. The search strategy incorporated Boolean operators and was applied uniformly across all platforms. The number of search results obtained per keyword from each platform is summarized in Table 1.

The literature review and data collection were conducted between August 2024 and March 2025. Scientific publications in English, Russian, Arabic, and Chinese were included in the review. The primary database searches and article screening were performed by Anastassiya Shevchenko and Akerke Amirkhanova. Other co-authors contributed by verifying the eligibility of the literature, extracting phytochemical and pharmacological data from selected manuscripts, and assisting with data synthesis and final categorization. The review process involved both gradual and parallel stages of search and screening to ensure coverage and accuracy. No conflicts arose during the literature review process.

We compared phytochemical composition data reported in selected studies to assess chemotypic variation across subspecies and plant organs. Specifically, we recorded identified metabolites, extraction solvents, analytical techniques (e.g., GC–MS, LC–MS/MS, NMR), and the corresponding plant parts and subspecies. Studies reporting distinct compound profiles from different subspecies (e.g., *A. nobilis* subsp. *neilreichii* vs. *A. nobilis* subsp. *nobilis*) or organs (e.g., aerial parts vs. flower heads) were reviewed comparatively to infer chemotypic differences. These patterns were synthesized to identify recurring subspecies-specific chemical signatures. The final synthesis was conducted by categorizing phytochemicals according to chemical class (e.g., flavonoids, terpenes, phenolic acids) and mapping them against plant parts and subspecies. This comparative approach allowed us to infer chemotypic variations and associate them with environmental or genetic factors, as reported by primary authors. Differences in extraction protocols and analytical techniques were also considered to contextualize the findings.

By applying the inclusion and exclusion criteria, papers that met the inclusion criteria were selected for further investigation and content assessment. The inclusion criteria encompassed peer-reviewed articles, biological/pharmacological studies, systematic reviews, and meta-analyses that specifically addressed *A. nobilis*, its phytochemical composition, pharmacological properties, and ecological significance. Conversely, the exclusion criteria involved omitting gray literature, extended abstracts, presentations, keynotes, book chapters, and inaccessible publications. Additionally, articles discussing *Achillea* species without specific information on *A. nobilis* were excluded, as they fell outside the scope of this review, which aims to define the status quo of medicinal and ethnobotanical significance (MES).

**Figure 2:**
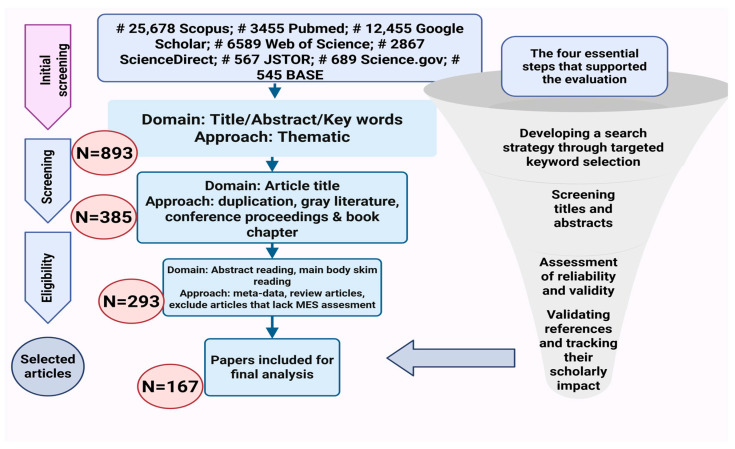
Schematic representation of the database search process for identifying and selecting publications in a systematic review (MES: medicinal and ethnobotanical significance).

The general screening process and the flow of selecting relevant literature are illustrated in Figure 2. Initially, approximately 28,000 records were retrieved from seven platforms. After eliminating non-relevant works, including gray literature, extended abstracts, presentations, keynotes, book chapters, and inaccessible sources, the number of retained articles was reduced to 893 for further title screening. Subsequently, 385 articles met the eligibility criteria for abstract evaluation. Following abstract screening, only 293 articles were deemed relevant for a full-text review. Among them, 167 studies specifically assessed MES, and these articles were downloaded for further detailed screening. Ultimately, 167 publications satisfied all inclusion criteria and were selected for the final analysis (Figure 2). The final set of relevant publications was then used for further evaluation and synthesis.

These terms facilitated the identification of studies related to the phytochemical composition, pharmacological potential, and ethnomedicinal applications of *A. nobilis*. Moreover, to enhance the accuracy of data retrieval, non-relevant materials were systematically excluded based on their lack of direct relevance to the study’s objectives. This methodological approach ensures the inclusion of high-quality and scientifically rigorous literature, thereby strengthening the reliability and validity of the findings. Additional studies were identified through manual screening of references in the selected articles. Furthermore, books containing high-quality taxonomic, ethnobotanical, and pharmacological information were also reviewed to ensure comprehensive coverage of *A. nobilis*. In addition to taxonomic and ethnobotanical aspects, particular attention was given to the pharmacological activities of the identified bioactive compounds. This approach provided a broader understanding of the therapeutic potential of *A. nobilis*, allowing for the identification of key bioactive constituents and their possible mechanisms of action. The literature reviewed spanned from 1952 to 2023.

The process of reviewing scientific articles to identify appropriate materials involves four key actions. First, selecting relevant keywords and developing a precise search strategy using Boolean operators ensures comprehensive database searches. Second, screening article titles and abstracts helps filter studies based on relevance and predefined inclusion/exclusion criteria. Third, full-text evaluation assesses methodological rigor, scientific credibility, and alignment with research objectives. Lastly, reference cross-checking and citation tracking enhance the review by identifying additional relevant studies through backward and forward citation analysis. This systematic approach ensures the selection of high-quality, scientifically relevant materials.

## 3. Distribution and Botanical Characterization

Based on botanical systematics, *Achillea* species belong to the Angiosperms, within the Eudicots clade, Campanulates order, Asteraceae family, Tubuliflorae subfamily, and Anthemideae tribe [37]. The *Achillea* genus comprises approximately 140 perennial herbaceous species worldwide [38]. These species are highly adaptable and thrive in diverse climates, particularly in semi-tropical, temperate, dry, and semi-arid regions [39]. They are commonly found in mountainous forests and steppes, exhibiting resilience in various soil conditions, including degraded lands and roadsides. Their adaptability is largely attributed to their ability to withstand moisture deficiency and direct sunlight, making them versatile in different environmental settings [37,40].

**Figure 3:**
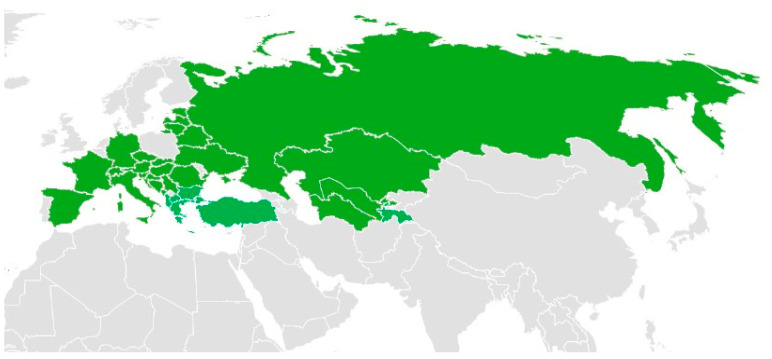
The distribution of *A. nobilis* (Green indicates the native distribution) [41]. The map was created using Microsoft Excel based on publicly available distribution data.

Among these species, *A. nobilis* is native to Europe, Asia, and parts of North Africa (Figure 3). Additionally, it has been reported to have been introduced into Great Britain, as well as several federal subjects of Russia, including Khabarovsk Krai, Primorye Krai, and Sakhalin. Moreover, its presence has been documented in the United States, specifically in Minnesota and Montana [41].

*A. nobilis*, commonly known as “noble yarrow”, is a perennial herbaceous plant reaching heights of 15–70 cm, with a rootstock that lacks stolons. Its stems are terete, longitudinally striped, and covered with spreading pilose hairs. The leaves are woolly-pubescent, with basal leaves being 2–3-pinnatipartite, oblong-lanceolate (2–10 × 1–3 cm) with a dentate rachis, while median cauline leaves are ovate-oblong to broadly ovate (2–5 × 1–3 cm), with primary segments regularly 1-pinnatifid to 1-pinnatisect. The species produces 50–150 capitula in dense corymbose inflorescences (2–10 cm wide), each capitulum being broadly obovoid to oblong (3–3.5 × 2–3 mm), with phyllaries ranging from triangular-lanceolate and acute to oblong and obtuse, covered in either sparse hairs or dense woolly-shaggy indumentum. The ligules are pale yellow above and white below (0.8–1.5(–2) mm), with 10–25 disk flowers per capitulum. Flowering occurs from June to August [42].

## 4. Uses of *A. nobilis* in Ethnomedicine

Plants constitute the principal healthcare resource in numerous communities globally [43]. They additionally function as vital sources for the advancement of biomedicines, and species traditionally utilized have made substantial contributions to the formulation of biomedical pharmaceuticals [44]. Statistical evidence indicates that traditional remedies, including phytotherapeutic agents, represent the most significant and at times the sole means of therapeutics for nearly 80% of the global population [45]. For example, approximately 85% of the global populace utilizes herbal medicines for both disease prevention and treatment, with demand rising in both developed and developing nations [46,47]. It is estimated that around 500 million individuals in South Asian countries alone seek health security through plant-based resources [44].

*A. nobilis *widely recognized for its medicinal properties, is one of the most notable examples of a plant extensively utilized in ethnomedicine across diverse cultures, where it has been traditionally employed for its anti-inflammatory, wound healing, and antimicrobial effects (Table 2) [48,49,50,51,52]. For instance, *A. nobilis* has been traditionally used in Bosnia and Herzegovina for treating bedwetting in children, blood purification, and various skin conditions, including injuries, rashes, and psoriasis. Moreover, its flower tincture has been widely applied, as it is believed to effectively restore the skin to its normal condition. This longstanding use highlights the plant’s significance in ethnomedicine for dermatological and internal health applications [48].

The seminal text *Shipagerlik Bayan* (“Confessions of a Healer”), authored by the esteemed Kazakh medical practitioner Oteyboydak Tleukabyl during the 15th century AD, provides a comprehensive exposition of the medical paradigms pertaining to pharmacology, anatomy, pathology, immunology, and nutritional care within the framework of Traditional Kazakh Medicine (TKM). Each of these paradigms constitutes a systematic framework comprising six principal components, specifically, kengistik tugyr (space), turak tugyr (earth), suwik tugyr (coldness), ystyk tugyr (heat), zharyk tugyr (light), and karangy tugyr (darkness) [53,54,55]. The foundations of ancient Kazakh folk medicine are rooted in the empirical observations and practical knowledge accrued over numerous generations. Traditionally, the array of medicinal flora utilized by the Kazakh populace was categorized into four primary classifications—fortifying, refreshing, warming, and laxative plants [55,56]—which were adeptly employed to address a variety of ailments, including bronchitis, bronchial asthma, urethritis, chronic rheumatoid arthritis, gastric discomfort, hyperacidity, diarrhea, hemostatic issues, metrorrhagia, envenomations from snake bites, and neoplastic diseases [56,57,58]. Among the diverse *Achillea* species, *A. nobilis* has been particularly noted within Kazakh folk medicine for its therapeutic application in treating urethritis and bronchitis. Additionally, its aqueous tincture has been employed in dermatological treatments for psoriasis [52].

## 5. Phytochemistry

Studying the phytoconstituents of *A. nobilis* is essential, particularly due to their pharmacological significance. However, a comprehensive literature survey indicates that the majority of phytochemical studies on *A. nobilis* employing various techniques and methodologies, were conducted between 1978 and 2020 (Table 3) [18,19,20,21,22,23,24,25,26,27,28,29,30,31,32,33,34]. This suggests a limited number of investigations dedicated to the plant’s chemical composition. Furthermore, an analysis of existing studies on *A. nobilis* and its subspecies (*A. nobilis* subsp. *neilreichii* and *A. nobilis* subsp. *sipylea*) reveals that most research has focused on water-based extracts. Additionally, studies have predominantly examined the aerial parts of the plant, with no reported investigations of the root composition. Consequently, *A. nobilis* and its subspecies exhibit a diverse array of phytochemicals, including phenolic compounds (flavonoids and flavonoid glycosides), terpenes (monoterpenes, oxygenated monoterpenes, sesquiterpenes), sesquiterpene lactones, guaianolides, and steroidal compounds (terpenoid derivatives).

Based on the literature, several compounds have been isolated from *Achillea nobilis* and its subspecies, including apigenin 7-glycoside [18], luteolin 7-glucoside [18], apigenin 7-glucuronide [18], the guaianolide estafiatin [19], hanphyllin [19], anobin [19], 3,5-dihydroxy-6,7,8-trimethoxyflavone [19], anolide [21], tanaparthin-β-peroxide [22], 5-hydroxy-3,6,7,4′-tetramethoxyflavone [22], chrysartemin A [22], canin [22,23], and chrysartemin [23]. These compounds were obtained through phytochemical isolation and characterized using standard techniques such as nuclear magnetic resonance (NMR) spectroscopy and mass spectrometry (MS). In comparison, the remaining compounds discussed in this review, which include phenolic compounds, terpenes and their derivatives, and other biologically active constituents, were identified based on analytical profiling or reported in the literature without direct isolation from *A. nobilis*.

Flavonoids are one of the primary categories of phytochemicals found in Achillea nobilis L. and its subspecies. Initial research by Solomko et al. [18] employing chromatographic and spectroscopic methods uncovered flavonoid glycosides such as apigenin-7-glucoside, luteolin-7-glucoside, and apigenin-7-glucuronide, from the non-flowering shoots of *A. nobilis* Follow-up studies by Adekenov et al. [19] and Krenn et al. [24] built upon these discoveries by recognizing flavonoid subclasses like 3,5-dihydroxy-6,7,8-trimethoxyflavone and quercetin-3-o-methyl ether from flower heads and aerial parts, respectively. In a more recent study, Taşkın et al. [33] revealed the existence of phenolic compounds like dicaffeoylquinic acid and several glycosylated derivatives of apigenin and luteolin in *A. nobilis* subsp. *neilreichii*, affirming the continued importance of flavonoids and phenolics in the phytochemistry of this group.

Various studies of *A. nobilis* and its subspecies have consistently reported monoterpenes, oxygenated monoterpenes, and sesquiterpene lactones. Palić et al. [25] discovered oxygenated monoterpenes, including *α*-thujone (25.7%) and borneol (9.9%), while Karamanendres et al. [26] and Ghani et al. [27] reported elevated concentrations of *α*-thujone, 1,8-cineole, and fragranol via GC–MS examinations of essential oils. Demirci’s research [28] further highlighted the prevalence of fragranol (24%) and *β-*eudesmol (8%) in the aerial parts. Additional research (e.g., Azizi et al. [30], Rustaiyan et al. [31], Kazemizadeh et al. [32]) verified the extensive occurrence of monoterpenes like cineole, camphor, and spathulenol. The headspace examination conducted by Ozdemir [34] similarly identified camphor and *α*-thujone as key volatile compounds. These terpenes are linked to various pharmacological effects, such as antimicrobial and anti-inflammatory properties.

The chemical makeup of *A. nobilis* differs not just by plant section but also by subspecies. For example, *A. nobilis* subsp. *neilreichii* exhibited a diverse range of monoterpenes and sesquiterpenes (e.g., Demirci [28], Ozdemir [34]), while *A. nobilis* subsp. *nobilis* and *A. nobilis* subsp. *neilreichii* were studied mainly for flavonoids and flavonoid glycosides (Valant-Vetschera [20]). These results indicate the presence of chemotypic variations among subspecies, probably shaped by genetic and environmental influences. These differences hold significant consequences for choosing suitable subspecies for medicinal or industrial uses.

A diverse array of extraction methods and solvents has been used to obtain secondary metabolites from *A. nobilis.* Previous research utilized comprehensive aqueous extraction (Solomko et al. [18]) and chloroform-based techniques (Adekenov et al. [19]), while subsequent studies embraced more focused methods, such as hydro-distillation (e.g., Ghani et al. [27], Kazemizadeh et al. [32]), headspace solid-phase micro-extraction (Ozdemir [34]), and ethanol-ethyl acetate solvent combinations (Taşkın et al. [33]). Analytical methods evolved over the decades, transitioning from UV and IR spectroscopy to more sophisticated techniques such as GC–MS, LC–MS/MS, and NMR spectroscopy. This methodological advancement indicates a wider movement towards high-resolution, multi-faceted characterization of plant metabolites.

Newer research from 2010 and later shows a distinct emphasis on thorough chemical profiling through contemporary methods. For example, Azizi et al. [30] and Rustaiyan et al. [31] conducted GC–MS analyses on the aerial components, pinpointing significant monoterpenes and sesquiterpenes. Kazemizadeh et al. [32] enhanced this method by examining both leaves and flowers, whereas Taşkın et al. [33] utilized LC–MS/MS to detect phenolic components. Ozdemir’s latest study [34] utilized HS–SPME/GC–MS for identifying volatile compounds in *A. nobilis* subsp. *neilreichii*. These investigations highlight a transition towards accurate, quantitative analysis of intricate phytochemical mixtures, with increasing attention on essential oils and phenolics.

Grasping the chemical makeup of *A. nobilis* and its subspecies has important consequences for the pharmaceutical sciences. Flavonoids such as apigenin and luteolin, found in various studies, display strong antioxidant, anti-inflammatory, and anticancer effects [54]. Likewise, monoterpenes like α-thujone and 1,8-cineole exhibit antimicrobial and neuroprotective properties, positioning them as potential candidates for therapeutic advancement [56]. Additionally, phenolic acids and their derivatives might play a role in cardiovascular protection and metabolic regulation [57]. Therefore, thorough phytochemical analysis bolsters the creation of evidence-backed herbal treatments and increases the significance of traditional medicinal flora in contemporary healthcare.

Although significant advancements have been made, there are still numerous gaps in the chemical profiling of *A. nobilis* and its subspecies. Interestingly, the plant’s roots remain unexplored, and the majority of research has combined aerial components (leaves, stems, and flowers) without conducting separate assessments. This restricts our comprehension of the biosynthetic abilities specific to organs. Studies indicate that various plant components can generate unique metabolite profiles as a result of different metabolic pathways and ecological roles [58]. Thus, upcoming research should focus on the separate examination of specific organs, such as stems, roots, and flowers, to completely clarify the phytochemical variation of the species.

To thoroughly investigate the chemical profile of *A. nobilis* and its subspecies, future studies should employ a multi-omics strategy that combines metabolomics, transcriptomics, and chemotaxonomy. Sophisticated methods like UHPLC–HRMS, MALDI–TOF imaging, and molecular networking may reveal new bioactive compounds. Moreover, investigations of seasonal and geographical variations should be carried out to assess the stability of chemotypes in different environments. Partnerships with pharmacologists and bioinformaticians will be crucial for connecting phytochemicals to particular bioactivities. These integrative studies will enhance the phytochemical profile of *A. nobilis* and promote its thoughtful use in pharmaceuticals and nutraceuticals.

### 5.1. Phenolic Compounds

Phenolic compounds, including phenolic acids and flavonoids, are widely recognized for their antioxidant and anti-inflammatory characteristics, which may confer protective effects against malignancies, metabolic disorders such as diabetes, and neurodegenerative conditions [59,60,61]. Moreover, flavonoids, classified as a subclass of phenolic compounds, exhibit an extensive array of pharmacological properties, encompassing antioxidant, anti-inflammatory, anticancer, and cardioprotective effects [62]. Flavonol glycosides possess analogous attributes and are particularly investigated for their antioxidant and anti-inflammatory capabilities [63]. Furthermore, they also demonstrate anti-inflammatory, anticancer, and cardiovascular protective properties [64]. These compounds, which are plentiful in the natural environment, present a promising pathway for the innovation of novel therapeutic agents (Table 4, Compounds **1**–**23**) [65,66,67,68,69,70,71,72,73,74,75,76,77,78,79,80,81,82,83,84,85,86,87,88].

Flavonoid glucuronides such as apigenin-7-O-glucuronide (Compound **11**) and luteolin-7-O-glucuronide (Compound **12**) derived from *A. nobilis* demonstrate strong anti-inflammatory and neuroprotective properties. Compound **11** suppresses the production of nitric oxide and tumor necrosis factor-alpha in LPS-activated macrophages, suggesting significant immunomodulatory capabilities [65]. Compound **12** modulates microglial polarization and enhances endogenous antioxidant pathways, offering antidepressant, neuroprotective, and anti-inflammatory benefits [66]. It also displays antibacterial and anticancer effects by inhibiting reactive oxygen species and biofilm formation while modulating critical pathways such as MET/AKT/mTOR [67].

**Figure 4:**
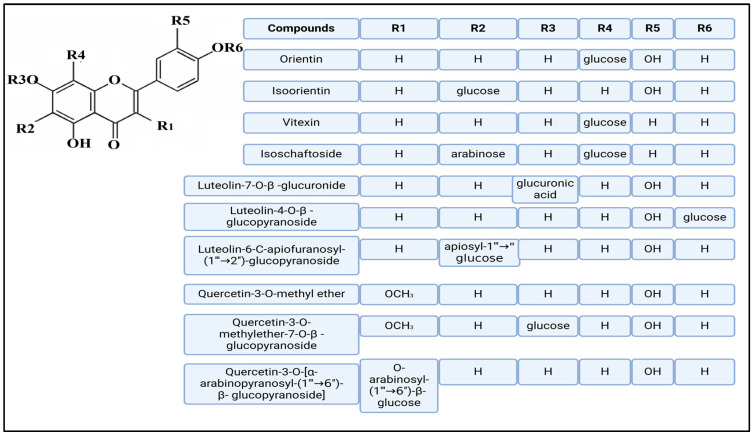
Chemical structures of selected flavone and flavonol glycoside derivatives identified in *Achillea nobilis* L. and its subspecies [24]. Compound **1**: orientin; Compound **2**: isoorientin; Compound **3**: vitexin; Compound **4**: isoschaftoside; Compound **5**: luteolin-7-O-β-glucuronide; Compound **6**: luteolin-4-O-β-glucopyranoside; Compound **7**: luteolin-6-C-apiofuranosyl-(1 → 2)-glucopyranoside; Compound **8**: quercetin-3-O-methyl ether; Compound **9**: quercetin-3-O-methylether-7-O-β-glucopyranoside; Compound **10**: quercetin-3-O-[α-arabinopyranosyl-(1 → 6)-β-glucopyranoside].

Methoxylated flavones, including 3,5-dihydroxy-6,7,8-trimethoxyflavone (Compound **13**) and 5-hydroxy-3,6,7,4′-tetramethoxyflavone (Compound **18**) from *A. nobilis* have shown enhanced anticancer effects, particularly when combined with 5-fluorouracil, resulting in reduced off-target toxicity in colon and pancreatic cancer treatments [71]. C-glycosylflavones such as vitexin (Compound **3**, Figure 4), isovitexin (Compound **14**), swertisin (Compound **15**), orientin (Compound **1**, Figure 4), and isoorientin (Compound **2**, Figure 4), isolated from *A. nobilis* subsp. *neilreichii*, exhibit a wide range of pharmacological effects. These include antioxidant, anti-inflammatory, neuroprotective, cardioprotective, antidiabetic, anticancer, hepatoprotective, and anxiolytic activities [72,73,74,75]. Swertiajaponin (Compound **16**), a C-methylated glycoside, has been implicated in the alleviation of metabolic syndromes such as hyperglycemia, hyperlipidemia, and insulin resistance [76], highlighting its therapeutic potential for metabolic disorders.

Flavonoids like quercetin-3-O-rhamnoside-7-O-glucoside (Compound 17) and quercetin-3-O-methylether-7-O-β-glucopyranoside (Compound **9**, Figure 4) demonstrate strong antioxidant activities. Quercetin-3-O-rhamnoside-7-O-glucoside (Compound **17**) inhibits polyphenol oxidase, preventing enzymatic browning, and enhances food antioxidant properties [77]. Quercetin-3-O-methylether-7-O-β-glucopyranoside (Compound **9**, Figure 4) shows protective effects against oxidative stress and in breast cancer models by inducing apoptosis and modulating PI3K/Akt and MAPK/Erk signaling pathways [82,83,84]. Luteolin (Compound **19**) and isorhamnetin-3-O-glucoside (Compound **20**) obtained from *A. nobilis* subsp. *neilreichii* also exhibit potent antioxidant, anti-inflammatory, and antiproliferative activities in endothelial and cancer cells, primarily through the STAT3 pathway and suppression of epithelial–mesenchymal transition (EMT) [85,86]. These compounds collectively enhance the plant’s medicinal potential through their multi-target bioactivities.

Among phenolic acids, dicaffeoylquinic acid (Compound **22**) and chlorogenic acid (Compound **23**) exhibit significant bioactivities. Compound **22** demonstrates antioxidant, anti-inflammatory, antimicrobial, cardioprotective, hepatoprotective, and antispasmodic properties, proving beneficial in the treatment of respiratory and metabolic disorders [87]. Compound **23**, an ester of hydroxycinnamic acid, has a wide range of pharmacological effects, including antibacterial, antiviral, anti-obesity, antihypertensive, antipyretic, and CNS-stimulating activities [88]. It also regulates lipid and glucose metabolism, making it useful in managing cardiovascular disease, diabetes, and obesity.

Several compounds such as luteolin-4-O-β-glucopyranoside (Compound **6**, Figure 4), luteolin-6-C-apiofuranosyl-(1”→2”)-glucopyranoside (Compound **7**, Figure 4), apigenin-6,8-di-C-glucoside (Compound **21**), and quercetin-3-O-[α-arabinopyranosyl-(1”→6”)-β-glucopyranoside] (Compound **10**, Figure 4) currently lack comprehensive pharmacological data (Figure 4). However, due to their structural similarity to well-characterized flavonoids such as luteolin and quercetin, they are hypothesized to possess antioxidant, anti-inflammatory, and neuroprotective activities. Studies have shown that C- and O-glycosylation enhance flavone stability and bioactivity, improving their modulatory effects on oxidative stress and inflammatory pathways [65,66,85]. Moreover, glycosidic modifications may enhance solubility and absorption, suggesting favorable pharmacokinetic profiles for future therapeutic applications [89,90,91,92,93,94,95,96,97,98,99,100,101,102,103].

### 5.2. Terpenes and Derivatives

A literature review revealed that *A. nobilis* and its subspecies, such as *A. nobilis* subsp. *sipylea* and *A. nobilis* subsp. *neilreichii*, contain a total of 157 terpene and terpene-derived compounds (Table 5). These substances are divided into various chemical categories: terpenes, terpenoids, sesquiterpenes, and derivatives of sesquiterpenoids. The chemical components were mainly extracted from different plant sections, including flower heads, aerial portions, and leaves, showcasing the plant’s abundant phytochemical diversity and the structural variety of its secondary metabolites. This wide range of terpenoid composition emphasizes the potential of *A. nobilis* and its subspecies as important sources of bioactive natural compounds.

A significant variety of terpenoids from *A. nobilis* display marked anti-inflammatory and antioxidant properties. For example, artemetin and caryophyllene oxide exhibit significant anti-inflammatory effects by blocking proinflammatory enzymes and mediators, which helps lower oxidative stress in multiple inflammatory models [105,112]. Carvacrol and thymol are crucial in managing inflammation by inhibiting NF-κB and MAPK pathways, and they have demonstrated effectiveness in decreasing oxidative damage, positioning them as promising options for treating inflammatory diseases [115,117]. Borneol exhibits synergistic anti-inflammatory properties and improves the bioavailability of concurrently administered anti-inflammatory agents [119]. These substances play a major role in the conventional application of *A. nobilis* for addressing inflammation-related ailments.

Multiple terpenes found in *A. nobilis* and its subspecies have demonstrated anticancer properties. Significantly, *β*-caryophyllene and caryophyllene oxide promote apoptosis and hinder cancer cell proliferation by affecting various signaling pathways, such as the PI3K/Akt axis [109,112]. Additionally, thymol and borneol have shown cytotoxic properties against several cancer cell lines, such as liver and breast cancer, with proof indicating their capability to induce cell cycle arrest and apoptosis [117,119]. Camphor, while mainly recognized for its antimicrobial properties, has also demonstrated potential cytostatic effects in cancer studies [120]. These results emphasize the therapeutic potential of *A. nobilis* terpenes in cancer prevention and therapy.

A significant amount of evidence backs the antimicrobial and antiviral properties of *A. nobilis* terpenes. Camphor, borneol, thymol, and carvacrol have shown significant antimicrobial properties against Gram-positive and Gram-negative bacteria, along with fungi [115,117,119]. Thymol and carvacrol specifically display membrane-disrupting characteristics that hinder microbial survival and biofilm development. Furthermore, sabinene and β-pinene have demonstrated antiviral properties by disrupting viral replication processes, aligning with the application of essential oils in traditional medicine for addressing respiratory and viral illnesses [113,114]. These terpenoids could function as useful substitutes for synthetic antimicrobial substances.

Certain terpene compounds derived from *A. nobilis* are noted to exert positive effects on the central nervous system. Thymol has been demonstrated to have anxiolytic and neuroprotective properties by influencing GABAergic neurotransmission and diminishing oxidative damage to neurons [117]. Borneol has shown notable neuroprotective effects, in part because it can traverse the blood–brain barrier and improve drug transport to the brain [119]. In a similar manner, *β*-caryophyllene engages with cannabinoid receptors and demonstrates anxiolytic, antidepressant, and neuroprotective effects [109]. These results indicate a possible function for *A. nobilis* terpenoids in the treatment of neurodegenerative diseases and mental health therapies.

Besides the primary therapeutic areas mentioned earlier, various terpenoids exhibit a range of bioactivities. For instance, thujone and sabinene have shown insecticidal and antifeedant properties that are beneficial in pest control [122]. Myrtenol and camphene show gastroprotective and antispasmodic properties, which could be helpful for gastrointestinal issues [110,121]. Additionally, α-pinene and β-pinene exhibit bronchodilatory and mucolytic properties, aiding their application in respiratory disorders [113]. While certain compounds like aristolone and germacrene D remain inadequately researched, their structural resemblance to active terpenes implies probable antioxidant and anti-inflammatory functions. These adaptable pharmacological characteristics endorse the multifunctional capabilities of *A. nobilis* terpenes in clinical and ethnopharmacological uses.

### 5.3. Other Biologically Active Compounds

Other biologically active compounds identified in *A. nobilis* and its subspecies include simple hydrocarbons, fatty alcohols, fatty acids, heterocyclic compounds, and simple ketones, all of which contribute to the plant’s diverse pharmacological potential (Table 6). Among these, 1-octadecanol (Compound **26**) stands out due to its antibacterial, anti-inflammatory, emollient, and mucosal-protective characteristics, which support its use in vaginal drug delivery systems and various mucosal therapeutic formulations [159,160]. Hexadecanoic acid (Compound **27**), commonly referred to as palmitic acid, has been shown to promote the growth of bone marrow mesenchymal stem cells and displays various pharmacological properties, such as antimicrobial, antioxidant, anti-inflammatory, anticancer, hypocholesterolemic, and skin-protective effects. This establishes it as a significant bioactive lipid for therapeutic advancements [161]. Likewise, dillapiol (Compound **29**) has demonstrated antimicrobial and antifungal properties, indicating its relevance in the development of antimicrobial medications and natural preservation methods [162]. Additional compounds extracted from *A. nobilis* also show specific pharmacological effects. For instance, isovaleric acid (Compound **30**) has been reported to improve ovariectomy-induced osteoporosis by suppressing osteoclast differentiation, suggesting its potential use in managing bone health after menopause [163]. This supports the inclusion of isovaleric acid among clinically significant bioactives for bone metabolism and osteoporosis-related conditions.

On the other hand, several compounds found in *A. nobilis* such as tricosane (Compound **24**), hexadecanol (Compound **25**), indipone (Compound **28**), adamantane-1,3-dimethyl (Compound **31**), 3-chloro-4-tert-butyl-6-phenylpyridazin (Compound **32**), and pentadecanone (Compound **33**), currently do not have documented pharmacological activity according to the existing literature. Although these compounds have not been extensively studied, their structural features and chemical classifications suggest potential biological activity. For example, alkanes such as tricosane and ketones like pentadecanone frequently occur in essential oils recognized for their insect-repellent or antimicrobial properties, while pyridazine derivatives are being explored for their anti-inflammatory and anticancer effects. Therefore, future bioactivity-oriented studies are needed to determine the medicinal relevance of these lesser-known compounds.

## 6. Pharmacological Effects Studies on *A. nobilis* and Its Subspecies

*Achillea nobilis* L. and its subspecies *(A. nobilis* subsp. *neilreichii* and *A. nobilis* subsp*. sipylea)* have garnered growing scientific interest owing to their extensive array of bioactive compounds and varied pharmacological characteristics, establishing them as a potential natural shield against a broad range of pathological issues, as demonstrated by their various pharmacological effects (Figure 4). Notably, the ethanolic extract of *A. nobilis* demonstrated significant anticonvulsant potential in preclinical models. In a comparative study using maximal electroshock (MES), pentylenetetrazole (PTZ), and strychnine nitrate (STN)-induced seizure models in rats, *A. nobilis* extract exhibited dose-dependent anticonvulsant effects, particularly at 200 and 300 mg/kg doses. Specifically, in the MES and PTZ-induced convulsion tests, the extract delayed the onset of seizures and reduced seizure duration, indicating central nervous system depressant activity. Moreover, at a dose of 300 mg/kg, *A. nobilis* provided 60% protection against STN-induced seizures, suggesting its potential role in modulating glycine-mediated neurotransmission. However, lower doses (100 and 200 mg/kg) did not show significant protection in the STN model, highlighting a possible threshold for efficacy. Consequently, these findings support the potential of *A. nobilis* as a natural source of anticonvulsant agents and warrant further investigation into its active constituents and mechanisms of action [164].

Another study revealed that the ethanol extract of *A. nobilis* subsp. *neilreichii* has noteworthy antinociceptive and anti-inflammatory effects, highlighting its possible therapeutic importance in managing pain and inflammation. The extract produced a significant antinociceptive response during the later phase of the formalin test at doses of 100, 200, and 400 mg/kg (i.p.), demonstrating its efficacy in alleviating inflammatory pain. Furthermore, it demonstrated anti-inflammatory effects in the carrageenan-induced paw edema model, especially at 100 and 200 mg/kg, indicating the suppression of acute inflammatory reactions. Nonetheless, no notable antinociceptive effect was noted in the tail-flick test, suggesting restricted effectiveness in altering spinal reflexes to thermal stimuli. Importantly, the administration of 400 mg/kg lengthened the latency on the hot-plate test at 60 and 90 min, indicating possible central analgesic effects without impairing sensory motor function. Additionally, the extract showed a significant safety margin, exhibiting an intraperitoneal LD_50_ value of 4456 mg/kg in mice. As a result, these results emphasize the anti-inflammatory and mild analgesic effects of *A. nobilis* subsp. *neilreichii*, endorsing its continued investigation in pain management studies [165].

Additionally, the research examined the antispasmodic properties of the total extract from *A. nobilis* subsp. *sipylea* on isolated rat duodenum, offering pharmacological support for its historical use in treating gastrointestinal spasms. The extract was assessed via a range of experiments that included cumulative dose–response curves for acetylcholine (ACh) and calcium chloride (CaCl_2_), conducted both without and with established spasmolytic agents like atropine, papaverine, and verapamil. The findings showed that the extract had a concentration-dependent inhibitory effect on contractions induced by ACh and CaCl_2_, noticeably decreasing the maximum contractile responses of the duodenal tissue. Significantly, this effect resembled that of papaverine and verapamil, but was different from atropine, indicating a mechanism of action that does not depend on muscarinic receptor antagonism and is more likely related to direct relaxation of smooth muscle or modulation of calcium channels. Additionally, the extract reduced K^+^-induced contractions, highlighting its muscle-relaxing properties. As a result, these results suggest that *A. nobilis* subsp. *sipylea* has significant antispasmodic properties, probably through the disruption of calcium influx and smooth muscle contractions, and may offer potential therapeutic benefits for gastrointestinal issues marked by hypermotility or spasms [166].

The protective benefits of *Achillea* species indigenous to Turkey, such as *A. nobilis* subspecies, have been studied regarding oxidative stress caused by hydrogen peroxide (H_2_O_2_) in human erythrocytes and leucocytes. This research assessed the antioxidant capacity of infusions made from 15 *Achillea* species, which are commonly used in traditional Turkish medicine, by measuring their effect on vital antioxidant enzymes—catalase (CAT), superoxide dismutase (SOD), and glutathione peroxidase (GPx)—along with lipid peroxidation (LPO) and glutathione (GSH) concentrations. The findings revealed that all *Achillea* infusions provided considerable protective benefits against oxidative damage induced by H_2_O_2_ by boosting the function of antioxidant defense systems in both erythrocytes and leucocytes. Significantly, *A. falcata* was the most potent in improving CAT, SOD, and GPx activities in red blood cells. Among the infusions tested, *A. crithmifolia* and *A. nobilis* subsp. *neilreichii* showed the greatest stimulation of CAT activity in leukocytes, while *A. millefolium* subsp. *pannonica* was the most effective for SOD, and *A. teretifolia* for GPx. Moreover, *A. nobilis* subsp. *sipylea* and *A. setacea* exhibited the most significant protective effects on LPO levels in leucocytes, while *A. millefolium* subsp. *pannonica* and *A. falcata* showed the least LPO levels in erythrocytes. As a result, these results indicate that *Achillea* species, especially the *A. nobilis* subspecies, serve as a valuable source of natural antioxidants that may be useful in the prevention and management of diseases linked to oxidative stress [167].

The essential oils extracted from *A. nobilis* subsp. *sipylea* and *A. nobilis* subsp. *neilreichii* show different levels of antibacterial and antifungal effects against various clinically significant microorganisms, with *A. nobilis* subsp. *sipylea* displaying the strongest antimicrobial capability. This subspecies exhibited significant antifungal properties against *Candida albicans*, resulting in an inhibition zone of 41 mm, and also demonstrated considerable antibacterial effects against *Escherichia coli*, *Staphylococcus aureus*, and *Salmonella typhimurium*, with inhibition zones varying from 12 to 15 mm. Conversely, essential oils obtained from *A. nobilis* subsp. *neilreichii*, gathered from various geographic sites, showed diminished antimicrobial properties; however, there was still some activity, especially against Gram-positive bacteria like *Staphylococcus epidermidis* and *S. aureus*. None of the essential oils evaluated were successful against *Pseudomonas aeruginosa*, renowned for its significant inherent resistance [27]. While the antimicrobial effectiveness of these essential oils was typically less than that of standard antibiotics like amoxicillin and sulbactam/ampicillin, the findings still indicate significant biological activity [141,164]. These results back the possible application of *A. nobilis*, especially subsp. *sipylea*, as a hopeful natural source of antimicrobial compounds, with regional differences in chemical makeup probably explaining variations in effectiveness [26,27]. Another study assessed the antibacterial properties of various extracts from *A. nobilis* subsp. *neilreichii* against *S. aureus* ATCC 25923, emphasizing how the extraction method and solvent type influence antimicrobial effectiveness. The Soxhlet extracts, especially those derived from ethyl acetate and chloroform, exhibited the strongest activity, showing minimum inhibitory concentrations (MICs) of 3.1 µg/mL and 3.87 µg/mL, respectively. Conversely, extracts obtained via ultrasonic bath with chloroform and maceration with n-hexane, chloroform, or ethanol exhibited lesser effects, resulting in MIC values surpassing 5 µg/mL and exceeding 6.25 µg/mL in certain instances. These findings indicate that Soxhlet extraction using semi-polar solvents improves the extraction of antibacterial compounds from the plant material. Furthermore, while the plant extracts were not as effective as the standard antibiotic meropenem (MIC, 0.012 µg/mL), they still suggest that *A. nobilis* subsp. *neilreichii* could be a valuable source of natural antibacterial compounds, especially when suitable extraction techniques are utilized [33].

While the cited pharmacological studies on *A. nobilis* and its subspecies provide valuable insights into their biological activities, several limitations should be acknowledged. Most experiments have been conducted using in vivo rodent models or basic in vitro assays, with limited mechanistic investigations to identify specific molecular targets. Additionally, many studies rely on crude extracts rather than purified compounds, which makes it difficult to determine which constituents are responsible for the observed effects. The sample sizes and control designs are sometimes insufficiently described, and variations in extraction methods may lead to inconsistent results across studies.

Importantly, a comprehensive literature review using multiple scientific databases (as detailed in the methodology section) found no published human clinical studies investigating *A. nobilis* or its subspecies. This significant gap indicates that the species is underexplored in clinical research, and highlights the need for rigorous human trials to validate its safety, efficacy, and therapeutic relevance. Furthermore, comprehensive toxicity and pharmacokinetic evaluations are lacking, and most studies do not assess long-term safety. These limitations underscore the need for standardized experimental protocols and advanced preclinical and clinical research to support the integration of *A. nobilis* and its bioactive components into evidence-based pharmaceutical and nutraceutical applications.

## 7. Conclusions

This review highlights the ethnomedicinal importance of *A. nobilis* and its subspecies (*A. nobilis* subsp. *neilreichii* and *A. nobilis* subsp. *sipylea*), which have been esteemed for ages in conventional medical practices. These plants have been extensively used to address a range of issues, such as inflammation, pain, infections, spasms, and convulsions. Their application in decoctions, oils, and infusions showcases the vibrant cultural history related to their therapeutic uses. Although there is extensive traditional usage, scientific investigation into their pharmacological mechanisms and bioactive components is still incomplete.

Existing phytochemical investigations show that *A. nobilis* comprises a variety of compounds, particularly flavonoids, flavonoid glycosides, monoterpenes, sesquiterpenes, sesquiterpene lactones, and terpenoid derivatives. These substances are associated with noteworthy pharmacological impacts, including antioxidant, antibacterial, antifungal, anti-inflammatory, antinociceptive, antispasmodic, central analgesic, and anticonvulsant effects. The existence of these bioactive compounds not only confirms traditional applications but also establishes the plant as a strong contender for drug development. Nonetheless, the majority of research has focused on water-based extracts and above-ground portions, with minimal emphasis on the chemistry specific to roots or variations within organs.

As indicated in the summarized pharmacological profile, *A. nobilis* and its varieties display a wide range of bioactivities. However, these effects differ based on subspecies, part of the plant, and extraction technique. Significant chemotypic variation is impacted by environmental and genetic influences, necessitating accurate chemotaxonomic and organ-specific evaluations. Incorporating multi-omics strategies, including metabolomics, transcriptomics, and sophisticated analytical methods (e.g., UHPLC–HRMS, MALDI–TOF), will be crucial for thoroughly charting the metabolite profile of this species and linking it to pharmacological results.

In summary, this review emphasizes the medical potential and overlooked research prospects related to *A. nobilis* and its subspecies. Future research should focus on examining underexplored plant components such as roots, assess seasonal and geographical impacts on phytochemical variations, and create solid pharmacological connections through interdisciplinary teamwork. Such initiatives will yield important understanding for the evidence-driven use of these plants in contemporary pharmacology and nutraceutical development.

## Figures and Tables

**Table 1:** The number of searches for each keyword.

Platforms	Keywords						Total
	*A. nobilis*	*A. nobilis* Phytochemicals	*A. nobilis* Compounds	*A. nobilis* Pharmacological	*A. nobilis* Traditional use	*A. nobilis* Ethnomedicine	
Scopus	30	0	0	0	0	0	30
Pubmed	17	1	8	5	1	1	33
Google Scholar	14,200	2380	3570	2100	3370	704	26,324
Web of Science	73	1	23	1	5	0	103
ScienceDirect	96	71	355	214	320	37	1093
JSTOR	121	0	13	0	7	0	141
Science.gov	202	12	224	170	146	8	762
BASE	433	1	58	13	16	2	523

“0” means not found.

**Table 2:** Ethnomedicinal use of *A. nobilis* in different cultures.

Country	Plant Part	Ethnomedicinal Use(s)	Ref.
Bosnia and Herzegovina	flower	managing nocturnal enuresis in children, cleansing the blood, and addressing a range of dermatological issues (wounds, irritations, and psoriasis)	[48]
Iran	above-ground part	antiparasitic, wound-healing and anti-infective agent	[49]
Turkey	above-ground part	abdominal pain and flatulence	[50]
China	root	treating hypertension	[51]
Kazakhstan	above-ground part	treating urethritis, bronchitis, and psoriasis	[52]

**Table 3:** Phytochemical composition studies of *A. nobilis* and its subspecies.

Year	Species	Plant of Part	Methods of Extraction	Extract	Techniques/Methods	Identified Chemical Classes	Major Compounds	Ref.
1978	*A. nobilis*	Non-Flowering Shoots	hydrodistillation	water	Chromatographic analysis, UV and IR spectra	flavonoids	apigenin-7-glycoside (cosmosiin), luteolin-7-glucoside (cynaroside)	[18]
1984	*A. nobilis*	Flower Heads	column chromatography	chloroform	IR, PMR, MS	terpenes, flavonoids	anobin, estafiatin, hanphyllin, 3,5-dihydroxy-6,7,8-trimethoxyflavone	[19]
1987	*A. nobilis* subsp. *nobilis*, *A. nobilis* subsp. *nedrelchll*	Leaves	N/S	N/S	TLC, UV, NMR	flavonoid glycosides	vitexin, orientin, isoorientin, di-C-glycosylapigenins, isovitexin, swertiajaponin, di-C-glycosylluteolins, C-glycosylflavone	[20]
1994	*A. nobilis*	Aerial Part	hydrodistillation	water	IR, UV, PMR, NMR, X-ray	sesquiterpene lactones	anolide	[21]
1995	*A. nobilis*	Flower Heads	column chromatography	N/S	MS, ^1^H-NMR, ^13^C-NMR, 2D-NMR	guaianolides, monoterpenes	tanaparthin-β-peroxide, 5-hydroxy-3,6,7,4′-tetramethoxyflavone	[22]
1999	*A. nobilis*	Aerial Part	aqueous extraction, silica gel chromatography	hexane-ethyl acetate	IR, PMR	sesquiterpene lactones	chrysartemin a, canin	[23]
2003	*A. nobilis*	Aerial Part	methanol extraction	methanol	UV, ^1^H-NMR, ^13^C-NMR, ESI–MS	flavonoids	luteolin-6-c-apiofuranosyl-(1′′′ --> 2′′)-glucoside, orientin, quercetin-3-o-methyl ether	[24]
2003	*A. nobilis*	Flower Heads	hydrodistillation	water	GC–MS	monoterpenes, oxygenated	α-thujone (25.7%), artemisia ketone (14.8%), borneol (9.9%) and camphor (8.2%)	[25]
2007	*A. nobilis* subsp*. sipylea*, *A. nobilis* subsp. *neilreichii*	Flower Heads	hydrodistillation	water	GC–MS	monoterpenes, oxygenated monoterpenes, sesquiterpenes	fragranol, dihydro-eudesmol, linalool, chrysanthenone	[26]
2008	*A. nobilis*	Aerial Part	hydrodistillation	N/S	GC, GC–MS	monoterpenes, sesquiterpenes	α-thujone (34.06%), 1,8-cineole (14.14%) and β-cedren epoxide (9.63%)	[27]
2009	*A. nobilis* subsp. *nobilis*, *A. nobilis* subsp. *neilreichii*	Aerial Part	hydrodistillation (Clevenger-type apparatus)	N/S	GC, GC–MS	monoterpenes, sesquiterpenes	fragranyl acetate (32%), fragranol (24%), and β-eudesmol (8%)	[28]
2010	*A. nobilis*	Aerial Part	hexane extraction, column chromatography	hexane	IR, PMR, ^13^C-NMR	steroidal compounds	acetyleucanbin	[29]
2010	*A. nobilis*	Aerial Part	hydrodistillation	dichloromethane	GC–MS	monoterpenes, sesquiterpenes	α-thujone, 1,8-cineole, artemisia ketone	[30]
2011	*A. nobilis*	Aerial Part	hydrodistillation	methanol	GC, GC–MS	oxygenated monoterpenes,	artemisia ketone, 1,8-cineole, yomogi alcohol, spathulenol	[31]
2011	*A. nobilis*	Leaves, Flowers	hydrodistillation (Clevenger-type apparatus)	N/S	GC, GC–MS	monoterpenes, sesquiterpenes	1,8-cineole, geranyl isovalerate, trans-verbenol	[32]
2018	*A. nobilis* subsp. *neilreichii* (Kerner)	Aerial Part	ethanol and ethyl acetate extraction	ethanol, ethyl acetate	HPLC–DAD, LC–MS/MS	phenolic compounds	dicaffeoylquinic acid, luteolin-7-o-glucoside, apigenin-6,8-di-c-glucoside	[33]
2019	*A. nobilis* subsp. *neilreichii*	Aerial Part	solid-phase micro-extraction	N/S	HS–SPME/GC–MS	monoterpenes, sesquiterpenes	camphor, germacrene d, 1,8-cineole, α-thujene	[34]

Here, UV: Ultraviolet Spectroscopy; IR: Infrared Spectroscopy; PMR: Proton Magnetic Resonance (another term for ^1^H-NMR); MS: Mass Spectrometry; TLC: Thin-Layer Chromatography; NMR: Nuclear Magnetic Resonance; ^1^H-NMR: Proton Nuclear Magnetic Resonance; ^13^C-NMR: Carbon-13 Nuclear Magnetic Resonance; 2D-NMR: Two-Dimensional Nuclear Magnetic Resonance; ESI-MS: Electrospray Ionization Mass Spectrometry; GC–MS: Gas Chromatography–Mass Spectrometry; GC: Gas Chromatography; HPLC-DAD: High-Performance Liquid Chromatography with Diode-Array Detection; LC–MS/MS: Liquid Chromatography–Tandem Mass Spectrometry; HS-SPME/GC-MS: Headspace Solid-Phase Microextraction Gas Chromatography–Mass Spectrometry; HPLC: High-Performance Liquid Chromatography; HS-SPME: Headspace Solid-Phase Microextraction; N/S: not specified.

**Table 4:** Phenolic compounds found in *A. nobilis* and its subspecies.

No.	Compounds	Plant Species	Investigated Plant Part	Chemical Class (Subclass)	Chemical Structure	Known Pharmacological Activities
1	Apigenin 7-glucuronide [18]	*A. nobilis*	Leaves/flowers	Flavonoid (Flavone glucuronide)	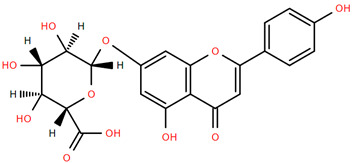 Compound **11**	significantly inhibited nitric oxide (NO) production in LPS-stimulated macrophages (RAW264.7) (at 1 μg/mL, NO inhibition was 30.7%; at 10 μg/mL, NO inhibition reached 97.1%); also reduced tumor necrosis factor-alpha (TNF-α) production (at 5 μg/mL, TNF-α inhibition was 26.2%, at 10 μg/mL, TNF-α inhibition was 83.8%) [65,68,69,70]
2	Luteolin 7-glucoside (cynaroside) [18]	*A. nobilis*	Leaves/flowers	Flavonoid (Flavone glycoside)	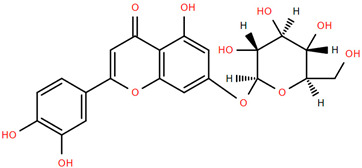 Compound **12**	exhibits antidepressant, anti-inflammatory, anti-ferroptotic, and neuroprotective effects by modulating microglial M1 polarization and the IRF1/SLC7A11/GPX4 signaling pathway in LPS-stimulated BV-2 cells and CUMS-induced mice [66]; shows antibacterial, antifungal, antileishmanial, antioxidant, hepatoprotective, antidiabetic, anti-inflammatory, anticancer, and anti-apoptotic activities, including inhibition of ROS, biofilm formation, and the MET/AKT/mTOR pathway [67]
3	3,5-Dihydroxy-6,7,8-Trimethoxyflavone [19]	*A. nobilis*	Leaves/flowers	Flavonol	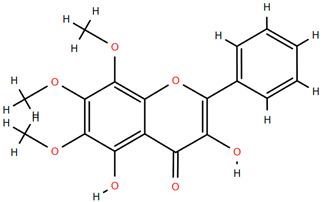 Compound **13**	synergizes with 5-fluorouracil, allowing for dose reduction and reduced off-target toxicity in the treatment of colonic and pancreatic cancers [71]
4	Vitexin [20,24]	*A. nobilis* subsp. *nobilis*, *A. nobilis* subsp. *neilreichii*	Leaves	Flavonoid (C-glycosylflavone	See Figure 3 Compound **3**	exhibits antioxidant, anti-inflammatory, neuroprotective, cardioprotective, anticancer, anxiolytic, antidiabetic, hepatoprotective, antimicrobial, antithyroid, analgesic, and anti-osteoporotic activities [72]
5	Isovitexin [20]	*A. nobilis* subsp. *nobilis*, *A. nobilis* subsp. *neilreichii*	Leaves	Flavonoid (C-glycosylflavone)	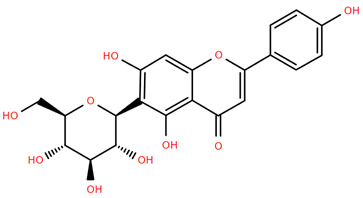 Compound **14**	exhibits antioxidant, anti-inflammatory, neuroprotective, anticancer, antidiabetic, cardioprotective, hepatoprotective, antimicrobial, and anti-anxiety activities [73]
6	Swertisin [20]	*A. nobilis* subsp. *nobilis*, *A. nobilis* subsp. *neilreichii*	Leaves	Flavonoid (C-glycosylflavone)	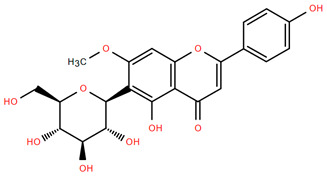 Compound **15**	exhibits antioxidant, anti-inflammatory, anticancer, hepatoprotective, antidiabetic, neuroprotective, and anti-obesity activities [74]
7	Orientin [20,24]	*A. nobilis* subsp. *nobilis*, *A. nobilis* subsp. *neilreichii*	Leaves	Flavonoid (C-glycosylflavone)	See Figure 3 Compound **1**	exhibits anticancer, antioxidant, neuroprotective, cardioprotective, antiallergic, and anti-inflammatory [75]
8	Isoorientin [20,24]	*A. nobilis* subsp. *nobilis*, *A. nobilis* subsp. *neilreichii*	Leaves	Flavonoid (C-glycosylflavone)	See Figure 3 Compound **2**	exhibits antioxidant and anti-inflammatory activities and plays a key role in ameliorating metabolic complications such as hyperglycemia, hyperlipidemia, and insulin resistance [76]
9	Swertiajaponin [20]	*A. nobilis* subsp. *nobilis*, *A. nobilis* subsp. *neilreichii*	Leaves	Flavonoid (C-methylated glycoside)	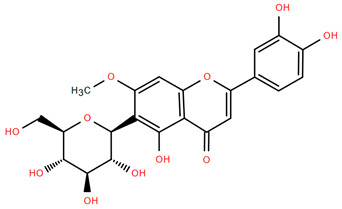 Compound **16**	exhibits potent antioxidant activity, inhibits enzymatic browning by inactivating polyphenol oxidase, and enhances the flavonoid content and antioxidant capacity of foods [77]
10	Quercetin 3-*O*-rhamnoside-7-*O*-glucoside [20]	*A. nobilis* subsp. *nobilis*, *A. nobilis* subsp. *neilreichii*	Leaves	Flavonoid (O-glycoside)	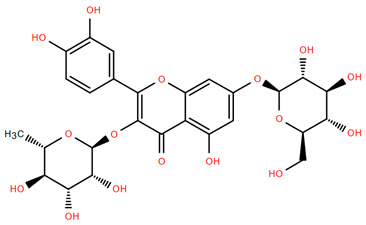 Compound **17**	possesses high anti-inflammatory activity by binding with interleukin-6 [78]
11	5-Hydroxy-3,6, 7,4′ -tetramethoxyfla- vone [22]	*A. nobilis*	Flower heads	Flavonoid (O-methylated flavone)	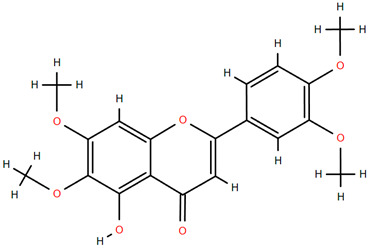 Compound **18**	known for its anticancer properties [79]
12	Isoschaftoside [24]	*A. nobilis*	Aerial part	Flavonoid (C-glycosylflavone)	See Figure 3 Compound **4**	inhibits lipopolysaccharide-induced inflammation in microglia through regulation of HIF-1 α-mediated metabolic reprogramming [80]; reverses nonalcoholic fatty liver disease via activating autophagy in vivo and in vitro [81]
13	Luteolin-7-*O*-*β*-glucuronide [24]	*A. nobilis*	Aerial part	Flavonoid (O-glucuronide)	See Figure 3 Compound **5**	NS
14	Luteolin-4-*O*-*β*-glucopyranoside [24]	*A. nobilis*	Aerial part	Flavonoid (O-glucoside)	See Figure 3 Compound **6**	NS
15	Luteolin-6-*C*-apiofuranosyl-(1′′′→2″)-glucopyranoside [24]	*A. nobilis*	Aerial part	Flavonoid (C,O-diglycoside)	See Figure 3 Compound **7**	NS
16	Quercetin-3-*O*-methyl ether [24]	*A. nobilis*	Aerial part	Flavonoid (O-methylated flavone)	See Figure 3 Compound **8**	protects FL83B cells from copper induced oxidative stress through the PI3K/Akt and MAPK/Erk pathway [82]; inhibits lapatinib-sensitive and -resistant breast cancer cell growth by inducing G (2)/M arrest and apoptosis [83]; protects FL83B cells from copper-induced oxidative stress through the PI3K/Akt and MAPK/Erk pathway [84]
17	Quercetin-3-*O*-methylether-7-*O*-*β*-glucopyranoside [24]	*A. nobilis*	Aerial part	Flavonoid (O-methylated glucoside)	See Figure 3 Compound **9**	NS
18	Quercetin-3-*O*-[α-arabinopyranosyl-(1′′′→6″)-*β*-glucopyranoside] [24]	*A. nobilis*	Aerial part	Flavonoid (Diglycoside)	See Figure 3 Compound **10**	NS
19	Luteolin	*A. nobilis* subsp. *neilreichii*	Aerial part [33]	Flavonoid (Flavone (aglycone))	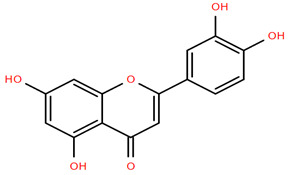 Compound **19**	blocks cancer development in vitro and in vivo by inhibition of proliferation of tumor cells, protection from carcinogenic stimuli, and activation of cell cycle arrest; reverses epithelial–mesenchymal transition (EMT) through a mechanism that involves cytoskeleton shrinkage, induction of the epithelial biomarker E-cadherin expression [86]
20	Isorhamnetin 3-O-glucoside	*A. nobilis* subsp. *neilreichii*	Aerial part [33]	Flavonoid (Flavonoid glycoside)	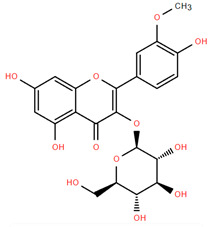 Compound **20**	NS
21	Apigenin-6,8-di-C-glucoside	*A. nobilis* subsp. *neilreichii*	Aerial part [33]	Flavonoid (Flavonoid glycoside)	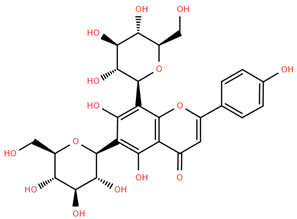 Compound **21**	NS
22	Dicaffeoylquinic acid	*A. nobilis* subsp. *neilreichii*	Aerial part [33]	Phenolic compound (Polyphenol (ester of caffeic acid and quinic acid))	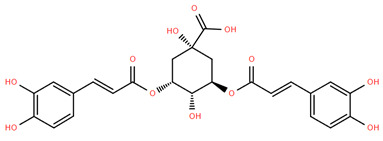 Compound **22**	exhibits antioxidant, anti-inflammatory, antimicrobial, hypoglycemic, cardioprotective, neuroprotective, hepatoprotective, antitussive, and antispasmodic activities, supporting their potential role in the treatment of respiratory diseases [87]
23	Chlorogenic acid	*A. nobilis* subsp. *neilreichii*	Aerial part [33]	Phenolic compound (Hydroxycinnamic acid ester (caffeic acid + quinic acid))	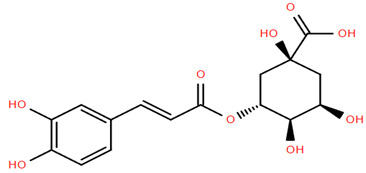 Compound **23**	exhibits a wide range of pharmacological activities, including antioxidant, anti-inflammatory, antibacterial, antiviral, neuroprotective, hepatoprotective, cardioprotective, antipyretic, anti-obesity, antihypertensive, antimicrobial, CNS-stimulating, hypoglycemic, and hypocholesterolemic effects, with significant roles in regulating lipid and glucose metabolism [88]

Here, “NS” means not studied.

**Table 5:** Terpenes and derivatives found in *A. nobilis* and its subspecies.

No.	Compounds	Plant Species	Investigated Plant Part	Chemical Class	Known Pharmacological Activities
1	Anobin [19]	*A. nobilis*	Leaves/flowers	Sesquiterpene lactone	NS
2	Estafiatin [19]	*A. nobilis*	Leaves/flowers	Sesquiterpene lactone	exerts anti-inflammatory effects on macrophages and protects mice from sepsis induced by LPS and cecal ligation puncture [104]
3	Anolide [21]	*A. nobilis*	Aerial part	Sesquiterpene lactone, anolide type	NS
4	4-Hydroperoxy-2*R*,3*R*-isopropyliden-hex-5-en-1-ol acetat [22]	*A. nobilis*	Flower heads	Acyclic monoterpenoid	NS
5	4-Hydroperoxy-2*S*,3*R*-isopropyli-den-hex-5-en-1-ol acetat [22]	*A. nobilis*	Flower heads	Acyclic monoterpenoid	NS
6	Tanaparthin-*β*-peroxide [22]	*A. nobilis*	Flower heads	Sesquiterpene lactone peroxide	NS
7	Clarysartemin [23]	*A. nobilis*	Aerial part	Sesquiterpene lactone	NS
8	Canin [23]	*A. nobilis*	Aerial part	Sesquiterpene lactone	NS
9	Sabinene [25,26,30]	*A. nobilis* [25,27,30], *A. nobilis* subsp. *sipylea*, *A. nobilis* subsp. *neilreichii* [26]	Aerial part [25,27], flower heads [26], leaves [30]	Bicyclic terpene	prevents skeletal muscle atrophy by Inhibiting the MAPK-MuRF-1 pathway in rats [105]
10	Yomogi alcohol [25,30,32]	*A. nobilis* [25,30,32]	Aerial part [25,32], leaves [30]	Monoterpene alcohol	NS
11	α-Terpinene [25,26,27,30,32]	*A. nobilis* [25,27,30,32], *A. nobilis* subsp. *sipylea*, *A. nobilis* subsp. *neilreichii* [26]	Aerial part [25,27,32], flower heads [26], leaves [30]	Monoterpene, cyclic diene	NS
12	*p*-Cymene [25,26,27,30,32]	*A. nobilis* [25,27,30,32], *A. nobilis* subsp. *sipylea*, *A. nobilis* subsp. *neilreichii* [26]	Aerial part [25,27,30,32], flower heads [26]	Monoterpene aromatic hydrocarbon	carvacrol and ρ-cymene established a strong synergistic antimicrobial effect against planktonic cultures of Gardnerella spp. [106]
13	1,8-Cineole [25,26,27,28,30,32]	*A. nobilis* [25,27,28,32], *A. nobilis* subsp. *sipylea*, *A. nobilis* subsp. *neilreichii* [26]	Aerial part [25,27,28,32], flower heads [26], leaves [30]	Cyclic terpene ether, oxide	shows anti-inflammatory and antioxidant mainly via the regulation on NF-κB and Nrf2 [107]
14	Artemisia ketone [25,32]	*A. nobilis*	Aerial part	Monoterpene ketone	NS
15	Artemisia alcohol [25,30,31,32]	*A. nobilis*	Aerial part [25,31,32], leaves [30]	Monoterpene alcohol	NS
16	α-Thujone [25,26,27,30,32]	*A. nobilis* [25,27,30,32], *A. nobilis* subsp. *sipylea*, *A. nobilis* subsp. *neilreichii* [26]	Aerial part [25,27,32], flower heads [26], leaves [30]	Monoterpene ketone, bicyclic	NS
17	*β-*Thujone [25,27,30]	*A. nobilis* [25,27]	Aerial part [25,27], Leaves [30]	Monoterpene ketone, bicyclic	NS
18	Camphor [25,26,27,30,32]	*A. nobilis* [25,27,30,32], *A. nobilis* subsp. *sipylea*, *A. nobilis* subsp. *neilreichii* [26]	Aerial part [25,27,32], flower heads [26], leaves [30]	Monoterpene ketone, bicyclic	potential anti-orthopoxvirus agent [108]
19	Pinocarvone [25,26,32,34]	*A. nobilis* [25,32,34], *A. nobilis* subsp. *sipylea*, *A. nobilis* subsp. *neilreichii* [26]	Aerial part [25,32,34], flower heads [26]	Monoterpene ketone	NS
20	Borneol [25,26]	*A. nobilis* [25], *A. nobilis* subsp. *sipylea*, *A. nobilis* subsp. *neilreichii* [26]	Aerial part [25], flower heads [26]	Monoterpene alcohol, bicyclic	potential anti-orthopoxvirus agent [108]
21	Terpinen-4-ol [25,26,28,30,31]	*A. nobilis* [25,28,30,31], *A. nobilis* subsp. *sipylea*, *A. nobilis* subsp. *neilreichii* [26]	Aerial part [25,28,31], flower heads [26], leaves [30]	Monoterpene alcohol	antibacterial and antibiofilm agent against *Staphylococcus aureus* [109]
22	α*-*Terpineol [25,26,27,30,31,32]	*A. nobilis* [25,27,30,31,32], *A. nobilis* subsp. *sipylea*, *A. nobilis* subsp. *neilreichii* [26]	Aerial part [25,27,31,32], flower heads [26], leaves [30]	Monoterpene alcohol	suppresses glioblastoma aggressive behavior and downregulates KDELC2 expression (anti-tumor) [110]
23	Myrtenal [25,34]	*A. nobilis*	Aerial part	Monoterpene aldehyde	shows antimicrobial, antifungal, antiviral, anticancer, anxiolytic, and neuroprotective properties [111]
24	Myrtenol [25,34]	*A. nobilis*	Aerial part	Monoterpene alcohol	shows anti-inflammatory, anticancer, antimicrobial effects [112]
25	*trans*-Carveol [25,31]	*A. nobilis*	Aerial part [25,31]	Monoterpene alcohol	NS
26	*cis*-Carveol [25]	*A. nobilis*	Aerial part	Monoterpene alcohol	NS
27	Cuminaldehyde [25]	*A. nobilis*	Aerial part	Terpene (Phenylpropanoid aldehyde)	ameliorates hyperglycemia [113]
28	Carvone [25]	*A. nobilis*	Aerial part	Monoterpene ketone	shows antimicrobial, antispasmodic, anti-inflammatory, antioxidant, antinociceptive, anticonvulsant [114]
29	Bornyl acetate [25,26,32]	*A. nobilis* [25,32], *A. nobilis* subsp. *sipylea*, *A. nobilis* subsp. *neilreichii* [26]	Aerial part [25,32], flower heads [26]	Monoterpene ester	a promising agent for immune modulation and inflammation [115]
30	Carvacrol [25]	*A. nobilis*	Aerial part	Monoterpene phenol	carvacrol and ρ-cymene established a strong synergistic antimicrobial effect against planktonic cultures of Gardnerella spp. [116]
31	Thymol [25]	*A. nobilis*	Aerial part	Monoterpene phenol	exhibits antimicrobial, antioxidant, anticarcinogenesis, anti-inflammatory, and antispasmodic activities [116]
32	Eugenol [25,26,28,32]	*A. nobilis* [25,28,32], *A. nobilis* subsp. *sipylea*, *A. nobilis* subsp. *neilreichii* [26]	Aerial part [2,25,32], flower heads [26]	Terpene (Phenylpropanoid (allylbenzene))	shows antibacterial; antifungal; antioxidant [117]
33	*trans*-Myrtenol acetate [25]	*A. nobilis*	Aerial part	Monoterpene ester	NS
34	*β*-caryophyllene [25,30,31,32]	*A. nobilis*	Aerial part [25,31,32], leaves [30]	Sesquiterpene hydrocarbon	exhibits anticancer and analgesic properties [118]
35	Linalool butyrate [25]	*A. nobilis*	Aerial part	Monoterpene ester	NS
36	(*E*)-*β*-farnesene [25]	*A. nobilis*	Aerial part	Sesquiterpene hydrocarbon, acyclic	NS
37	*β*-selinene [25,31]	*A. nobilis*	Aerial part [25,31]	Sesquiterpene hydrocarbon, bicyclic	NS
38	*β*-himachalene [25]	*A. nobilis*	Aerial part	Sesquiterpene hydrocarbon, tricyclic	NS
39	*δ*-Cadinene [25,28,31,32]	*A. nobilis*	Aerial part [25,28,31,32]	Sesquiterpene hydrocarbon	inhibits the growth of ovarian cancer cells [119]
40	Ledol [25,32,34]	*A. nobilis*	Aerial part [25,32]	Sesquiterpene alcohol, tricyclic	NS
41	Spathulenol [25,28,30,32,34]	*A. nobilis*	Aerial part [25,28,32,34], leaves [30]	Sesquiterpene alcohol	NS
42	Viridiflorol [25,26]	*A. nobilis* [25], *A. nobilis* subsp. *sipylea*, *A. nobilis* subsp. *neilreichii* [26]	Aerial part [25], flower heads [26]	Sesquiterpene alcohol, tricyclic	inhibits the growth of ovarian cancer cells [120]
43	α-eudesmol [25]	*A. nobilis*	Aerial part	Sesquiterpene alcohol, tricyclic	NS
44	α-Cadinol [25,27]	*A. nobilis*	Aerial part [25,27]	Sesquiterpene alcohol, tricyclic	NS
45	Patchouli alcohol [25]	*A. nobilis*	Aerial part	Sesquiterpene alcohol	demonstrates diverse pharmacological activities, including antiviral, antidepressant, antinociceptive, vasorelaxant, lung- and neuroprotective, anti-ulcer, anti-colitis, prebiotic-like, anti-inflammatory, anticancer, and metabolic disease-protective effects [121]
46	*β*-bisabolol [25,26,28]	*A. nobilis* [25,28], *A. nobilis* subsp. *sipylea*, *A. nobilis* subsp. *neilreichii* [26]	Aerial part [25,28], flower heads [26]	Sesquiterpene alcohol, acyclic	NS
47	*trans*-α-Bergamotol [25]	*A. nobilis*	Aerial part	Sesquiterpene alcohol, monocyclic	NS
48	α-Thujene [26,30,32]	*A. nobilis* subsp. *sipylea*, *A. nobilis* subsp. *neilreichii* [26], *A. nobilis* [30,32]	Flower heads [26], leaves [30], aerial part [32]	Monocyclic monoterpene hydrocarbon	NS
49	α-Pinene [26,30,32]	*A. nobilis* subsp. *sipylea*, *A. nobilis* subsp. *neilreichii* [26], *A. nobilis* [30,32]	Flower heads [26], leaves [30], aerial part [32]	Bicyclic monoterpene hydrocarbon	antitumor agent for the treatment of T-cell tumors [122,123]
50	Camphene [26,27,30,32]	*A. nobilis* subsp. *sipylea*, *A. nobilis* subsp. *neilreichii* [26], *A. nobilis* [27,30,32]	Flower heads [26], aerial part [27,32], leaves [30]	Bicyclic monoterpene hydrocarbon	NS
51	*β*-Pinene [26,27,28,30,32]	*A. nobilis* subsp. *sipylea*, *A. nobilis* subsp. *neilreichii* [26], *A. nobilis* [27,28,30,32]	Flower heads [26], aerial part [27,28,32], leaves [30]	Bicyclic monoterpene hydrocarbon	exhibits antibiotic resistance, anticoagulant, antitumor, antimicrobial, antimalarial, antioxidant, anti-inflammatory, anti-Leishmania, and analgesic properties [123]
52	Myrcene [26]	*A. nobilis* subsp. *sipylea*, *A. nobilis* subsp. *neilreichii*	Flower heads	Acyclic monoterpene hydrocarbon	exhibits anxiolytic, antioxidant, anti-ageing, anti-inflammatory, analgesic properties [124]
53	α-Campholenal [31]	*A. nobilis*	Aerial part [31]	Monoterpene aldehyde (bicyclic)	NS
54	Limonene [26]	*A. nobilis* subsp. *sipylea*, *A. nobilis* subsp. *neilreichii*	Flower heads	Monocyclic monoterpene hydrocarbon	antioxidant, antidiabetic, anticancer, anti-inflammatory, cardioprotective, gastroprotective, hepatoprotective, immune modulatory, anti-fibrotic, anti-genotoxic [125]
55	*γ*-Terpinene [26,27,30,32,34]	*A. nobilis* subsp. *sipylea*, *A. nobilis* subsp. *neilreichii* [26,34], *A. nobilis* [27,30,32]	Flower heads [26], aerial part [27,32,34], leaves [30]	Monocyclic monoterpene hydrocarbon	NS
56	Tricyclene	*A. nobilis* subsp. *neilreichii*	Aerial part [34]	Tricyclic monoterpene hydrocarbon	NS
57	δ-3-Carene	*A. nobilis* subsp. *neilreichii*	Aerial part [34]	Bicyclic monoterpene hydrocarbon	NS
58	Terpinolene [26,30,32,34]	*A. nobilis* subsp. *sipylea*, *A. nobilis* subsp. *neilreichii* [26,34], *A. nobilis* [30,32]	Flower heads [26], leaves [30], aerial part [32]	Monocyclic monoterpene hydrocarbon	NS
59	*trans*-Sabinene hydrate [30,32]	*A. nobilis*	Leaves [30], Aerial part [32]	Monocyclic monoterpene hydrocarbon	NS
60	*cis*-Sabinene hydrate [26,30,32]	*A. nobilis* subsp. *sipylea*, *A. nobilis* subsp. *neilreichii* [26], *A. nobilis* [30,32]	Flower heads [26], leaves [30], aerial part [32]	Monocyclic monoterpene hydrocarbon	NS
61	Linalool [26,28,32]	*A. nobilis* subsp. *sipylea*, *A. nobilis* subsp. *neilreichii* [26], *A. nobilis* [28,32]	Flower heads [26], aerial part [28,32]	Acyclic monoterpene alcohol	exhibits sedative, anxiolytic, antimicrobial, anti-inflammatory, antinociceptive, and anticancer effects [126]
62	Chrysanthenone [26]	*A. nobilis* subsp. *sipylea*, *A. nobilis* subsp. *neilreichii*	Flower heads	Bicyclic monoterpene ketone	NS
63	Camphene hydrate [26]	*A. nobilis* subsp. *sipylea*, *A. nobilis* subsp. *neilreichii*	Flower heads	Bicyclic monoterpene alcohol	NS
64	Fragranol [26,28]	*A. nobilis* subsp. *sipylea*, *A. nobilis* subsp. *neilreichii* [26], *A. nobilis* [28]	Flower heads [26], aerial part [28]	Monoterpene alcohol (isomer of linalool)	NS
65	Tetrahydro-linalool acetate [26]	*A. nobilis* subsp. *sipylea*, *A. nobilis* subsp. *neilreichii*	Flower heads	Saturated monoterpene ester (acetate of a linalool derivative)	NS
66	Piperitone [26]	*A. nobilis* subsp. *sipylea*, *A. nobilis* subsp. *neilreichii*	Flower heads	Monoterpene ketone	NS
67	Sabinyl acetate [26]	*A. nobilis* subsp. *sipylea*, *A. nobilis* subsp. *neilreichii*	Flower heads	Monoterpene acetate ester	exhibits antioxidant, antimicrobial, and anti-implantation activities [127]
68	Geranyl acetate [26,31]	*A. nobilis* subsp. *sipylea*, *A. nobilis* subsp. *neilreichii* [26], *A. nobilis* [31]	Flower heads [26], aerial part [31]	Acyclic monoterpene ester	antibacterial, anti-inflammatory, and larvicidal activity; inhibits enzymes involved in bacterial cell wall synthesis [128]
69	p-Menth-1-en-8-ol [32]	*A. nobilis*	Aerial part	Monocyclic monoterpene alcohol	NS
70	Lavendulol [27]	*A. nobilis*	Aerial part	Monoterpene alcohol (acyclic or monocyclic)	antibacterial, insecticidal, and anti-inflammatory effects [129]
71	Terpinene-4-ol [27,32]	*A. nobilis*	Aerial part	Monocyclic monoterpene alcohol	antibacterial, antibiofilm, anti-inflammatory, anticancer, and antifungal activities
72	Cuminyl aldehyde [27,34]	*A. nobilis*	Aerial part	Aldehyde derivative of aromatic monoterpene	[130]
73	*cis*-Chrysanthenyl acetate [28]	*A. nobilis*	Aerial part	Monoterpene ester (acetate of chrysanthenol)	antidiabetic, antimicrobial, anti-inflammatory, and anticancer properties [131]
74	Lavandulyl acetate [28,30,31,32]	*A. nobilis*	Aerial part [28,32], leaves [30,31]	Acyclic monoterpene ester	NS
75	4-Terpinenyl acetate [28]	*A. nobilis*	Aerial part	Monocyclic monoterpene ester	NS
76	Fragranyl formate [28]	*A. nobilis*	Aerial part	Ester of fragranyl alcohol	NS
77	Fragranyl acetate [28]	*A. nobilis*	Aerial part	Ester of fragranyl alcohol	NS
78	Fragranyl isobutyrate [28]	*A. nobilis*	Aerial part	Ester of fragranyl alcohol	NS
79	Fragranyl butyrate [28]	*A. nobilis*	Aerial part	Terpenoid derivative	NS
80	Fragranyl 2-methylbutyrate [28]	*A. nobilis*	Aerial part	Terpenoid derivative (Ester of fragranyl alcohol)	NS
81	Fragranyl 3-methylbutyrate [28]	*A. nobilis*	Aerial part	Terpenoid derivative (Ester of fragranyl alcohol)	NS
82	Fragranyl valerate [28]	*A. nobilis*	Aerial part	Terpenoid (Monoterpene ester)	NS
83	Fragranyl hexanoate [28]	*A. nobilis*	Aerial part	Terpenoid (Ester of fragranyl alcohol and hexanoic (caproic) acid)	NS
84	Artemisiaketone [30]	*A. nobilis*	Leaves	Monoterpene ketone	antimicrobial and antifungal activities [132]
85	*cis*-Sabinol [30]	*A. nobilis*	Leaves	Monocyclic monoterpene alcohol (oxygenated sabinene)	exhibits antibacterial and antifungal properties [133]
86	Lavandulol [30,31]	*A. nobilis*	Leaves [30], aerial part [31]	Acyclic monoterpene alcohol	NS
87	Bornylacetate [30]	*A. nobilis*	Leaves	Bicyclic monoterpene ester	anti-inflammatory, analgesic, anticoagulant, and hepatoprotective activities [134,135]
88	Lavandulyl propionate [30]	*A. nobilis*	Leaves	Acyclic monoterpene ester	NS
89	Lavandulyl isobutanoate [30]	*A. nobilis*	Leaves	Acyclic monoterpene ester	NS
90	Lavandulyl-isovalerate [30]	*A. nobilis*	Leaves	Acyclic monoterpene ester	NS
91	Longipinocarvone [30]	*A. nobilis*	Leaves	Oxygenated bicyclic monoterpene	exhibits antimicrobial and cytotoxic properties [136]
92	Veridiflorol	*A. nobilis*	Aerial part [32]	Acyclic monoterpene ester	demonstrates antibacterial, antifungal, anti-inflammatory, and cytotoxic effects [137]
93	Neryl acetate	*A. nobilis*	Aerial part [31]	Acyclic monoterpene ester	Shows anti-inflammatory, antifungal, and neuroprotective activities [138,139]
94	Bornyl angelate	*A. nobilis*	Aerial part [31]	Bicyclic monoterpene ester	NS
95	Linalool oxide	*A. nobilis*	Aerial part [32]	Acyclic monoterpene epoxide	anxiolytic, anticonvulsant, and antinociceptive effects [140]
96	α-Cyclocytral	*A. nobilis*	Aerial part [32]	Monoterpene aldehyde	NS
97	p-Mentha-2-en-1-ol	*A. nobilis*	Aerial part [32]	Monocyclic monoterpene alcohol	NS
98	Isicyclocytral	*A. nobilis*	Aerial part [32]	Monoterpene aldehyde (isomer of cyclocitral)	NS
99	*trans*-3(10)-Carene-4-ol	*A. nobilis*	Aerial part [32]	Bicyclic monoterpene alcohol	NS
100	*cis*-Verbenol	*A. nobilis*	Aerial part [32]	Bicyclic monoterpene alcohol	antibacterial and acaricidal activity [141]
101	*trans*-Verbenol	*A. nobilis*	Aerial part [32]	Bicyclic monoterpene alcohol	antimicrobial and insecticidal activity [142]
102	Lavandolul	*A. nobilis*	Aerial part [32]	Acyclic monoterpene alcohol	NS
103	4-Terpineol	*A. nobilis*	Aerial part [32]	Monocyclic monoterpene alcohol	antifungal, antibacterial, and anti-inflammatory properties [143]
104	2-Pinene-10-ol	*A. nobilis*	Aerial part [32]	Bicyclic monoterpene alcohol	NS
105	*E*-3(10)Caren-2-ol	*A. nobilis*	Aerial part [32]	Bicyclic monoterpene alcohol	NS
106	*trans*-Crystantenyl acetate	*A. nobilis*	Aerial part [32]	Monoterpene ester	NS
107	*cis-*Crystantenyl acetate	*A. nobilis*	Aerial part [32]	Monoterpene ester	NS
108	Geranyl isovalerate	*A. nobilis*	Aerial part [32]	Acyclic monoterpene ester	NS
109	Lavandulyl acetate	*A. nobilis*	Aerial part [32]	Acyclic monoterpene ester	antimicrobial and sedative properties [144]
110	Limonene-6-ol-pivalate	*A. nobilis*	Aerial part [32]	Monoterpene ester	NS
111	Geranyl propionate	*A. nobilis*	Aerial part [32]	Acyclic monoterpene ester	NS
112	*β*-Bisabolene [26]	*A. nobilis* subsp. *sipylea*, *A. nobilis* subsp. *neilreichii*	Flower heads	Acyclic sesquiterpene hydrocarbon)	anti-inflammatory, antimicrobial, and anticancer activities [145]
113	γ-Cadinene [26]	*A. nobilis* subsp. *sipylea*, *A. nobilis* subsp. *neilreichii*	Flower heads	Bicyclic sesquiterpene hydrocarbon	antioxidant and anti-inflammatory effects [146]
114	Bicyclogermacrene [27,34]	*A. nobilis*	Aerial part	Bicyclic sesquiterpene hydrocarbon	antibacterial, anti-inflammatory, and cytotoxic activities [147]
115	*trans-β*-Guaiene [27]	*A. nobilis*	Aerial part	Bicyclic sesquiterpene hydrocarbon	NS
116	α*-*Copaene [28]	*A. nobilis*	Aerial part	Bicyclic sesquiterpene hydrocarbon	antifungal and cytotoxic effects [148]
117	*cis-*α-Bergamotene [28]	*A. nobilis*	Aerial part	Monocyclic sesquiterpene hydrocarbon	anti-inflammatory and insecticidal activities [149]
118	Sesquisabinene [28]	*A. nobilis*	Aerial part	Tricyclic sesquiterpene hydrocarbon	NS
119	γ-Curcumene [28]	*A. nobilis*	Aerial part	Aromatic sesquiterpene hydrocarbon	anti-inflammatory and anticancer properties [150]
120	ar-Curcumen [28]	*A. nobilis*	Aerial part	Aromatic sesquiterpene hydrocarbon	NS
121	Santolina triene [30,32]	*A. nobilis*	Aerial part	Tricyclic sesquiterpene hydrocarbon with three double bonds	NS
122	Germacrene D [30,32,34]	*A. nobilis*	Leaves [30], aerial part [32,34]	Bicyclic hydrocarbon	antimicrobial, anti-inflammatory, and cytotoxic properties [151]
123	α-Selinene	*A. nobilis*	Aerial part [31,34]	Eudesmane-type sesquiterpene hydrocarbon	NS
124	(*E*,*E*)*-*α-Farnesene	*A. nobilis*	Aerial part [31]	Acyclic sesquiterpene hydrocarbon	antifungal, and anti-inflammatory effects [152]
125	Geranyl isobutyrate [31,32]	*A. nobilis*	Aerial part [31,32]	Acyclic monoterpene ester	NS
126	α-Calacorene	*A. nobilis*	Aerial part [31]	Tricyclic sesquiterpene hydrocarbon	antimicrobial and antioxidant properties [153]
127	*β*-Chamigrene	*A. nobilis*	Aerial part [32]	Tricyclic sesquiterpene hydrocarbon	antimicrobial and cytotoxic activities [153]
128	Cedrene	*A. nobilis* subsp. *neilreichii*	Aerial part [34]	Bicyclic sesquiterpene hydrocarbon	NS
129	*β*-Eudesmol [26,28,31]	*A. nobilis* subsp. *sipylea*, *A. nobilis* subsp. *neilreichii* [26], *A. nobilis* [28,31]	Flower heads [26], aerial part [28,31]	Oxygenated sesquiterpene alcohol	NS
130	Dihydro-eudesmol [26]	*A. nobilis* subsp. *sipylea*, *A. nobilis* subsp. *neilreichii*	Flower heads	Saturated form of eudesmol	NS
131	α-Bisabolol [26]	*A. nobilis* subsp. *sipylea*, *A. nobilis* subsp. *neilreichii*	Flower heads	Oxygenated sesquiterpene alcohol	NS
132	Germacrene D-4-ol [27,28]	*A. nobilis*	Aerial part	Sesquiterpene alcohol	NS
133	Cedrene-*β-*epoxide [27]	*A. nobilis*	Aerial part	Epoxide derivative of cedrene	NS
172	Helifolenol [27]	*A. nobilis*	Aerial part	Oxygenated sesquiterpene	NS
134	*trans*-Sesquisabinene hydrate [28]	*A. nobilis*	Aerial part	Hydroxylated sesquiterpene	NS
135	Caryophyllene oxide [28,30,32,34]	*A. nobilis*	Aerial part [28,32,34], leaves	Epoxidized sesquiterpene	NS
136	Salvial-4(14)-en-1-one [28]	*A. nobilis*	Aerial part	Ketone-type sesquiterpenoid	NS
137	*cis*-Sesquisabinene hydrate [28]	*A. nobilis*	Aerial part	Hydroxylated sesquiterpene	NS
138	*γ*-Eudesmol [28,30]	*A. nobilis*	Aerial part, leaves	Sesquiterpene alcohol	NS
139	Eremoligenol [28]	*A. nobilis*	Aerial part	Hydroxylated sesquiterpene	NS
140	ar-Turmerol [28]	*A. nobilis*	Aerial part	Aromatic sesquiterpene alcohol	NS
141	neo-Intermedol [30]	*A. nobilis*	Leaves	Hydroxylated sesquiterpene	NS
142	Intermedeol [28]	*A. nobilis*	Aerial part	Guaiane-type sesquiterpene alcohol)	NS
143	Torilenol [28]	*A. nobilis*	Aerial part	Hydroxylated sesquiterpene	NS
144	Eudesma-4(15),7-dien-4β-ol [28]	*A. nobilis*	Aerial part	Eudesmane-type sesquiterpene alcohol	NS
145	*γ*-Costol [28]	*A. nobilis*	Aerial part	Oxygenated sesquiterpene	NS
146	Cadin-4-en-7-ol [30]	*A. nobilis*	Leaves	Sesquiterpenoid (Cadinane-type alcohol)	NS
147	Epi-α-Cadino	*A. nobilis*	Aerial part [31]	Sesquiterpenoid (Cadinane-type sesquiterpene alcohol)	NS
148	Selin-11-en-4-α-ol	*A. nobilis*	Aerial part [31]	Hydroxylated selinane-type sesquiterpene	NS
149	Khusinol	*A. nobilis*	Aerial part [31]	Oxygenated sesquiterpene alcohol	NS
150	Aristolone	*A. nobilis*	Aerial part [31]	Tricyclic sesquiterpene ketone	antimicrobial and anti-inflammatory activities [154]
151	Nerolydol	*A. nobilis*	Aerial part [32]	Acyclic sesquiterpene alcohol	antimalarial, antiparasitic, antifungal, and antioxidant effects [155]
152	Murolan-3,9(11)-diene-10peroxy	*A. nobilis*	Aerial part [32]	Oxygenated sesquiterpene peroxide	NS
153	Cubenol	*A. nobilis*	Aerial part [32]	Tricyclic sesquiterpene alcohol	antifungal, cytotoxic, and antioxidant activities [156]
154	3β-Cadin-4-en-10-o	*A. nobilis*	Aerial part [32]	Sesquiterpenoid (Cadinane-type sesquiterpene alcohol)	NS
155	Aromadenderen epoxide	*A. nobilis*	Aerial part [32]	Oxygenated sesquiterpene	antibacterial and anti-inflammatory activities [157,158]
156	Eudesm-7(11)-en-4-ol	*A. nobilis*	Aerial part [32]	Eudesmane-type sesquiterpene alcohol	NS
157	Hanphyllin [19]	*A. nobilis*	Leaves/flowers	Sequiterpene (Sesquiterpene lactones, germacranolide)	NS

Here, “NS” means not studied.

**Table 6:** Other biologically active compounds found in *A. nobilis* and its subspecies.

No.	Compounds	Plant Species	Investigated Plant Part	Chemical Class	Chemical Structure	Known Pharmacological Activities
1	Tricosane	*A. nobilis* subsp. *neilreichii*	Aerial part [34]	Alkane	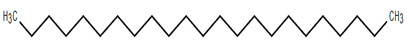 Compound **24**	NS
2	Hexadecanol	*A. nobilis*	Aerial part	Fatty alcohol	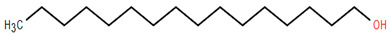 Compound **25**	NS
3	1-Octadecanol	*A. nobilis*	Aerial part	Fatty alcohol	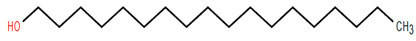 Compound **26**	exhibits antibacterial, anti-inflammatory, emollient, and mucosal-protective properties, supporting its use in vaginal drug-delivery hydrogels [159,160]
4	Hexadecanoic acid	*A. nobilis*	Aerial part [30]	Fatty acid	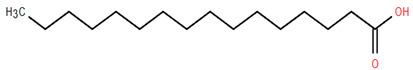 Compound **27**	induces proliferation of bone marrow mesenchymal stem cells, antimicrobial, antioxidant, anti-inflammatory, anticancer, hypocholesterolemic, and skin-protective effects [161]
5	Indipone	*A. nobilis*	Leaves [30]	Aromatic ketone	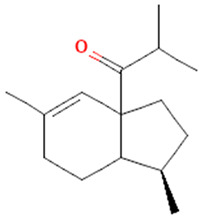 Compound **28**	NS
6	Dillapiol	*A. nobilis*	Aerial part [31]	Phenylpropanoid	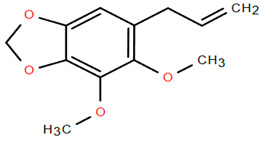 Compound **29**	antimicrobial and antifungal [162]
7	Isovaleric acid	*A. nobilis*	Aerial part [32]	Carboxylic acid	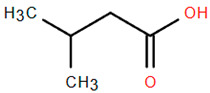 Compound **30**	ameliorates ovariectomy-induced osteoporosis by inhibiting osteoclast differentiation [163]
8	Adamantane,1,3-dimethyl	*A. nobilis*	Aerial part [32]	Polycyclic hydrocarbon	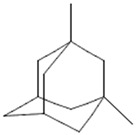 Compound **31**	NS
9	3-chloro-4-t-buthyl-6-phenyl pyridazin	*A. nobilis*	Aerial part [32]	Heterocyclic compound	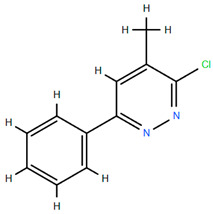 Compound **32**	NS
10	Pentadecanone	*A. nobilis* subsp. *neilreichii*	Aerial part [34]	Aliphatic ketone	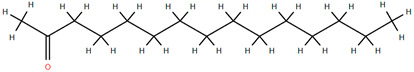 Compound **33**	NS

Here, “NS” means not studied.

## Data Availability

No new data were created or analyzed in this study. Data sharing is not applicable to this article.
